# Relative genetic diversity of the rare and endangered *Agave shawii* ssp. *shawii* and associated soil microbes within a southern California ecological preserve

**DOI:** 10.1002/ece3.7172

**Published:** 2021-01-22

**Authors:** Jeanne P. Vu, Miguel F. Vasquez, Zuying Feng, Keith Lombardo, Sora Haagensen, Goran Bozinovic

**Affiliations:** ^1^ Boz Life Science Research and Teaching Institute San Diego CA USA; ^2^ Southern California Research Learning Center National Park Services San Diego CA USA; ^3^ University of California San Diego Extended Studies La Jolla CA USA; ^4^ Biological Sciences University of California San Diego La Jolla CA USA

**Keywords:** 16S rRNA, biodiversity, conservation, fitness, microbiome, phylogenetics

## Abstract

Shaw's Agave (*Agave shawii* ssp. *shawii*) is an endangered maritime succulent growing along the coast of California and northern Baja California. The population inhabiting Point Loma Peninsula has a complicated history of transplantation without documentation. The low effective population size in California prompted agave transplanting from the U.S. Naval Base site (NB) to Cabrillo National Monument (CNM). Since 2008, there are no agave sprouts identified on the CNM site, and concerns have been raised about the genetic diversity of this population. We sequenced two barcoding loci, *rbcL* and *matK*, of 27 individual plants from 5 geographically distinct populations, including 12 individuals from California (NB and CNM). Phylogenetic analysis revealed the three US and two Mexican agave populations are closely related and have similar genetic variation at the two barcoding regions, suggesting the Point Loma agave population is not clonal. Agave‐associated soil microbes used significantly more carbon sources in CNM soil samples than in NB soil likely due to higher pH and moisture content; meanwhile, soil type and soil chemistry analysis including phosphorus, nitrate nitrogen, organic matter, and metals revealed significant correlations between microbial diversity and base saturation (*p* < 0.05, *r* ^2^ = 0.3676), lime buffer capacity (*p* < 0.01, *r* ^2^ = 0.7055), equilibrium lime buffer capacity (*p* < 0.01, *r* ^2^ = 0.7142), and zinc (*p* < 0.01, *r* ^2^ = 0.7136). Soil microbiome analysis within the CNM population revealed overall expected richness (*H*′ = 5.647–6.982) for *Agave* species, while the diversity range (1 − *D* = 0.003392–0.014108) suggests relatively low diversity marked by high individual variation. The most prominent remaining US population of this rare species is not clonal and does not seem to be threatened by a lack of genetic and microbial diversity. These results prompt further efforts to investigate factors affecting *Agave*'s reproduction and fitness.

## Graphical abstract

We performed phylogenetic analysis among five populations of endangered Agave populations by sequencing two barcoding genes, *rbcL* and *matK*. 16S rRNA V4 region sequencing was utilized to assess agave‐associated microbial population diversity within two sites, and microbial enzymatic activity assays were performed to quantify carbon source utilization as an indicator of functional diversity. Our results revealed similar genetic variation between the US and Mexican populations, suggesting that the US agave population in southern California preserve is not clonal, despite low effective population size and habitat fragmentation.

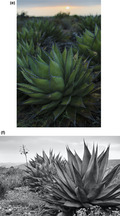

## 1 INTRODUCTION

**FIGURE 1:**
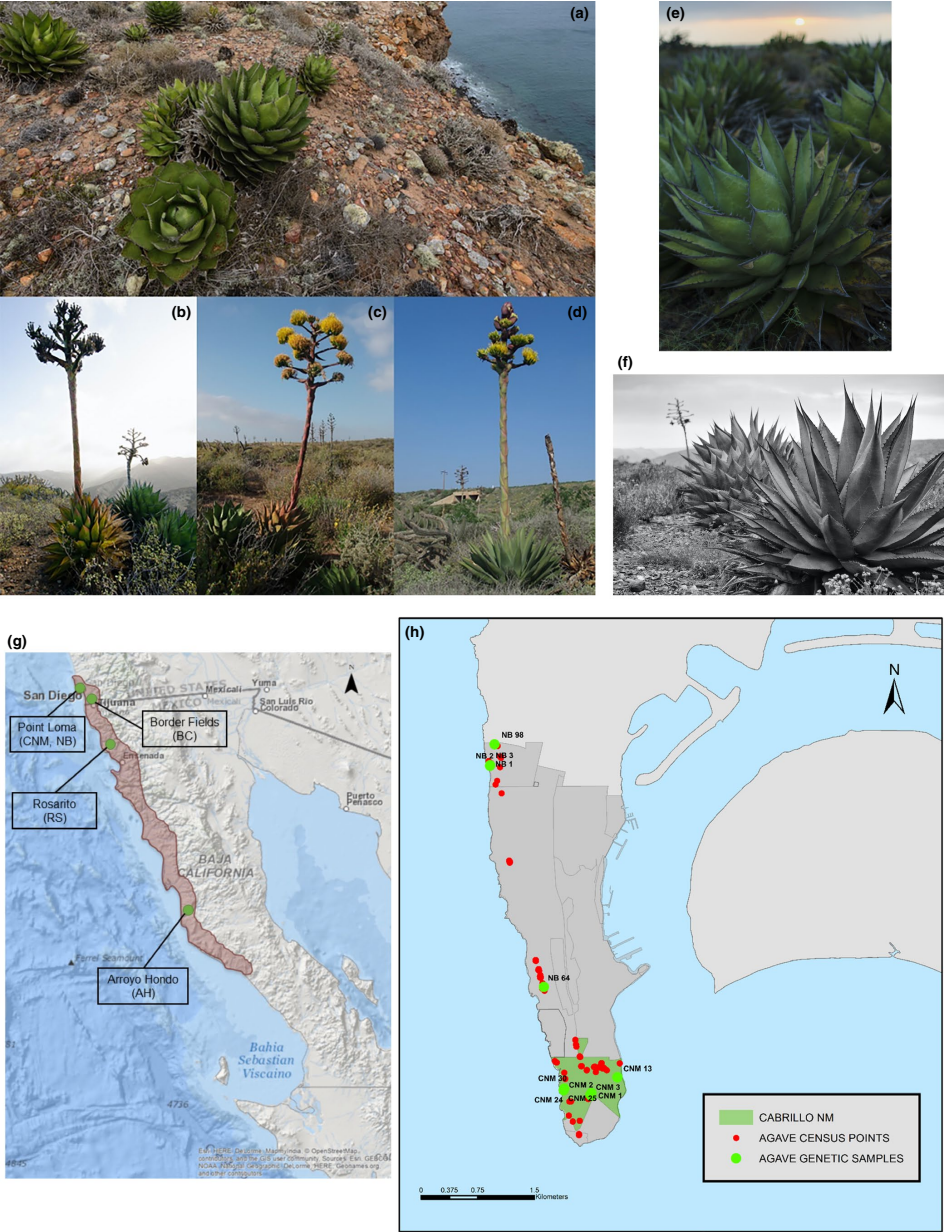
*Agave shawii* ssp. *shawii* rosettes across Cabrillo National Monument (US), Rosarito (MX), and Arroyo Hondo (MX). (a) Rosettes positioned adjacent to an eroding cliff at Cabrillo National Monument (CNM). Rosettes grow clonally, packed in clusters or as lone rosettes. Courtesy of Michael Ready. Flowering Shaw's Agave from (b) CNM, (c) Rosarito (RS), and (d) Arroyo Hondo (AH). Courtesy of Michael Ready and Sula Vanderplank. (e, f) Shaw's Agave in Valle Tranquilo Reserva Natural, San Quintin, northern Baja California. Courtesy of Michael Ready. (g) Habitat range of Shaw's Agave. Sites sampled in this study (Point Loma, CA; Border Fields, CA; Rosarito, MX; Arroyo Hondo, MX) are indicated with arrows. The image was generated in ArcGIS 10. (h) Sampling sites across Point Loma. A map of Shaw's Agave individuals in CNM and NB, generated in ArcGIS 10. Green circles denote sampled individuals used for genetic diversity analysis; red circles indicate individuals present in Point Loma but not sampled for genetic analysis. The CNM area is highlighted in green; the remaining peninsula (gray) is part of the NB.

Habitat loss and fragmentation are major threats to ecosystem stability. Habitat loss reduces the resources and area available to species, while habitat fragmentation results in patches of populations and limits interactions among them (Pardini et al., 2018). Both negatively affect biodiversity (Cagnolo et al., 2009; Newbold et al., 2015), food‐web structure (Bartlett et al., 2016; Cagnolo et al., 2009), and the dispersal and reproduction of species (Browne & Karubian, 2018; Lander et al., 2019; Torrenta et al., 2018). Slow growth and low reproductive rates species, such as agaves, are especially susceptible to environmental disturbances (Martínez‐Palacios et al., 1999). *Agave shawii* ssp. *shawii* (Shaw's Agave) is a maritime semelparous succulent, small‐to‐medium plant inhabiting the Pacific Coast from southwestern California to Baja California (Figure 1g). It has green ovate leaves 20–50 cm long and 8–20 cm wide, and spines along the margins of the leaves; rosettes grow 0.08–2 m wide and 1–2.5 m tall (Vanderplank, 2014; Vanderplank & Lombardo, 2015). It reproduces and grows slowly, taking 20–40 years to reach the flowering stage (Gentry, 1978). This taxon is seriously endangered in California, with a global rank between G2 (imperiled) and G3 (vulnerable), and a state rank of S1 (critically imperiled; California Native Plant Society, 2020).

Once abundant across coastal southern San Diego, the Shaw's Agave population has drastically declined since the 1970s primarily due to urban development (Gentry, 1978; Roof, 1971; Vanderplank, 2014). The population remnants consisting of native individuals and transplants over the last several decades exist on the northern edge of Naval Base Point Loma (NB) (Beauchamp, 1976; Gentry, 1978; Vanderplank, 2014; Vanderplank & Lombardo, 2015). Since 1976, Shaw's Agave threatened by eroding cliffs was transplanted from the NB into the neighboring Cabrillo National Monument (CNM) and across coastal San Diego County (Beauchamp, 1976; Vanderplank, 2014). Three total populations of Shaw's Agave in San Diego County were reported in 1978 (Gentry, 1978). In 2008, a native population on the east side of the Border Field State Park (Border Crossing, BC) was reduced to a single clump of rosettes due to the border fence construction. As of 2014, there are six total populations of Shaw's Agave in the United States: four entirely transplanted (CNM, Torrey Pines State Reserve), and two partially transplanted (east of BC and NB). The majority of rosettes survived transplantation (Vanderplank, 2014), but the transplantations may have compromised the plant's ecological fitness. Since 2008, no new germinating individuals of Shaw's Agave have been noted (Vanderplank, 2014; Vanderplank & Lombardo, 2015) and since 2014, fewer than 1,000 individuals grow in San Diego County. The lack of new seedlings, geographic isolation, and translocation contributed to a concern that the individuals at Point Loma are clonal. Mexican agave populations are more contiguous, intact, abundant, have a larger range of natural habitat, and have evidence of seedling recruitment (sexual reproduction) and thus presumably more genetically diverse; US agave populations grow in shallow soil, produce fewer rosettes per individual, and are considered to be almost exclusively clonal (Vanderplank et al., 2014; Vanderplank & Lombardo, 2015).

In this study, the relative genetic diversity within and between five Shaw's Agave populations from CNM (US), NB (US), BC (US), Rosarito, northern Baja California (RS‐MX), and Arroyo Hondo, northern Baja California (AH‐MX) populations was evaluated. We utilized DNA barcodes to confirm the species identity of these US agave populations with unclear transplantation origin at the molecular level and assess whether the observed differences between the US and MX populations are due to the US population being mostly clonal or having relatively lower genetic diversity. The standard DNA barcode genes for land plants, *rbcL* and *matK*, were sequenced to assess the relative genetic diversity of Shaw's Agave along its range. *rbcL* is a highly conserved gene in plants that offers high universality and high sequence quality but low species discrimination power, while *matK*, a rapidly evolving gene region, allows for high levels of species discrimination. The combination of these loci yields high resolution and discrimination power (Hollingsworth et al., 2009).

Microbial diversity is a biomarker for a plant's health and fitness (Srivastava et al., 1996). Plant fitness depends on inherited genes and microbes living on and in the organism (Rosenberg et al., 2010). Microbiota significantly contribute to plant health by promoting plant growth, nutrient cycling, suppression of soilborne plant diseases (Mendes et al., 2011; Podila et al., 2009; Rosenberg et al., 2010) and can increase plant fitness by limiting abiotic stress, including temperature, drought, and flooding (Grover et al., 2011). A recently transplanted individual may not have established a diverse and stable microbiome compared to older individuals, and soil composition was also noted as possibly affecting microbial diversity. Shaws' Agave‐associated soil samples, ranging from rocky, fibrous to fine sandy soil, were collected in CNM and NB (Figure 1h) to quantify soil microbiota diversity and activity via 16S rRNA sequencing and microbial enzymatic assays. The highly conserved 16S rRNA V4 gene region offers an effective survey of microbial diversity and microbial phylogenies assembly. Coupled with 16S rRNA sequencing data, the metabolic activity assay provides insight into the microbiome's structure, abundance, and robustness, and a comprehensive view of soil microbe living conditions. In this study, Biolog EcoPlates were utilized to determine the amount and identity of carbon sources used by the soil microbes. The sequence variation of the 16S rRNA V4 region served as an indicator of population diversity, while enzymatic carbon source utilization was an indicator of functional diversity. The moisture, pH, nutrients, and metals of soil surrounding Shaw's Agave individuals near CNM and the NB were correlated with genetic and microbial diversity to better understand the variables affecting species fitness.

A lack of genetic diversity among Shaw's Agave could leave the population vulnerable to disease, drought, and other stressors (Frankham et al., 2002; Keller & Waller, 2002). Species loss has negative ecological effects on predatorial food sources, parasitic or symbiotic organisms, and even the surrounding landscape (Holt & Loreau, 2002). Urbanization and U.S.–Mexico border fencing have led to the fragmentation of the *Agave*'s natural environment, potentially facilitating inbreeding and reducing gene flow. Considering its long reproductive cycle and habitat destruction (e.g., NB, BC), Shaw's Agave population may not recover naturally (Vanderplank, 2014). Due to no evidence of new growth, including lack of individual plant germination, at the southern California preserve for at least 12 years and no records indicating origin of transplanted plants, we hypothesized that fitness of the plants may be compromised by habitat fragmentation, low genetics diversity (clonal origin of transplanted *Agave*), low effective population size, and compromised soil microbial diversity. In this study, we assessed genetic relatedness and relative diversity comparing individual plants to others within and among five agave populations and quantifying soil microbe diversity of the individual plants within the southern California preserve.

Better understanding of intrinsic factors will help implement long‐term management plans. For instance, a lack of genetic diversity can be mitigated by introducing stock or pollen from Baja into the US population; an unhealthy microbiome could be improved with probiotics, or compromised sites can be flagged as unsuitable habitat. If no significant problems were identified, the conservation plan should focus on extrinsic factors, such as the activities of pollinators and herbivores on the US Shaw's Agave habitat.

## 2 MATERIALS/METHODS

### 2.1 Sampling

Shaw's Agave leaf tissues were collected from 27 individual plants (Figure 1g,h) across the habitat range (Figure 1g), that is, Point Loma (CNM, NB), Border Field State Park, Rosarito, and Arroyo Hondo. An individual is one tightly grouped cluster of rosettes (Figure 1a–c) or a lone rosette (Figure 1d). Individual's samples were collected from three sites in California, USA: CNM, CA (7; June 2017); NB, CA (5; June 2017); and BC, CA (5; March 2008), and two sites in Mexico: AH, MX (5; October 2008) and RS, MX (5; June 2008). Samples were collected from U.S. National Park Service (NPS)‐predetermined sites at CNM and NB. Leaf tissue surfaces were cleaned with 70% ethanol and excised with sterile scissors. Three replicates from each individual's rosette (Figure 1h) were collected at CNM and NB, placed on ice, and stored at −80°C. Leaf tissues collected from BC, AH, and RS (Figure 1g) were stored in silica gel.

Soil samples were collected at a maximal depth of 30 cm within 1 m from the center of Shaw's Agave rosettes following the root system lines and within 5 cm of peripheral roots at CNM and NB using ethanol‐treated aluminum coring devices. The rhizosphere was not sampled to avoid disturbing the shallow root system of these endangered plants. Three soil replicates per agave cluster were obtained within 2 m of each other and placed on ice prior to storage at −20°C.

### 2.2 Barcoding

Approximately 100 mg of Shaw's Agave spineless leaf tissue were snap‐frozen with liquid nitrogen and homogenized using a mortar and pestle. DNA was isolated using a DNeasy 96 Plant kit (Qiagen DNeasy) according to the manufacturer's instructions. Barcoding regions of *rbcL* and *matK* were amplified in an Eppendorf Mastercycler using GoTaq Green. Primers generated in Primer3plus for *rbcL* and *matK* are *rbcL*‐F 5′‐CTGCGAATTCCCCCTGCTTA‐3′, *rbcL*‐R 5′‐GATCGCGTCCCTCATTACGA‐3′, *matK*‐F 5′‐CAAAAGAGGTTCGTTGGGCA‐3′, and *matK*‐R 5′‐ATTGGCCCAGATCGGCTTAC‐3′. PCR products were purified using a PCR clean‐up kit (LAMDA Biotech), and samples were sequenced by Eton Bioscience Inc.

### 2.3 Phylogenetic tree construction

DNA sequences and chromatograms for *rbcL* and *matK* were visualized in FinchTV for quality checking. Multiple sequence alignments were performed in GUIDANCE2; DNA segments with low confidence and/or ambiguous reads were manually removed. Phylogenetic trees were generated in ClustalOmega using neighbor‐joining and edited in the Interactive Tree of Life (iTOL; Letunic & Bork, 2019).

### 2.4 Characterization of soil pH, moisture, and chemistry

pH: 3 g of each soil sample was diluted in 6 ml of sterile deionized water, vortexed, incubated at room temperature for 30 min, and gently vortexed every 5 min before pH was measured.

Moisture: 1 g soil samples were oven‐dried with a Quincy Lab Inc [QC] Model 30GC Lab Oven at 110°C for 7 days. Mass of each sample before and after incubation was compared to determine percent moisture content.

Chemical properties: Pooled soil samples from 12 sites (from CNM and NB) were sent to the Soil, Plant and Water (SPW) Laboratory at University of Georgia, where they were tested for lime buffer capacity (LBC) by measuring the pH before and 30 min after Ca(OH)_2_ addition (Sonon et al., 2015), pH (Kissel & Vendrell, 2012), cation exchange capacity (CEC), metallic elements, phosphorus, nitrate nitrogen by Mehlich I sum and Percent Base Saturation (Sonon et al., 2017), and organic matters (OM) by percentage weight loss on ignition for 3 hr at 360°C.

### 2.5 Characterization of soil functional biodiversity

Soil microbes were isolated and diluted into an EcoPlate to measure microbial metabolism over time. 0.5 g of soil was diluted in 14.5 ml of sterile water and vortexed at maximum speed for 5 min. Soil particles settled for 2 min, and a 1:30 dilution of the supernatant was added to one section of a 96‐well Biolog EcoPlate. *Escherichia coli* OP‐50 (Caenorhabditis Genetic Center, University of Minnesota, St. Paul, MN) was used as the positive control. Absorbances at 590 nm were recorded and compared before and after 7 days of incubation at 25°C. Negative values and changes less than 0.25 Abs were considered not biologically relevant and were set to zero (Garland, 1997). Wells were normalized to the maximal observed value for each carbon source. Functional biodiversity was quantified by calculating Shannon evenness and richness (Gryta et al., 2014).

### 2.6 16S rRNA microbial sequencing and analysis

Genomic DNA was extracted from soil samples using a Quick‐DNA Fecal/Soil Microbe kit (Zymo Research) per manufacturer's instructions. Library preparation was performed using the NEXTflex™ 16S V4 Amplicon‐Seq Kit 2.0. Libraries were quantified with a Bioanalyzer (Agilent 2100 and Agilent 2200 TapeStation) and pooled in a MiSeq flow cell for 100,000 reads per sample for 36 samples (12 sites, Point Loma). 16S rRNA sequences (~7 million) were imported and analyzed using Mothur software (version 1.39.5; Kozich et al., 2013) toolset on Galaxy (usegalaxy.org; Afgan et al., 2018). Forward and reverse paired sequences were aligned and grouped by sample sites. Sequences were aligned to the SILVA 132 bacterial reference (Quast et al., 2013; Yilmaz et al., 2014). Sequences aligned to region 10,357–25,452 in the SILVA reference were selected for further analysis and denoized by the pre.cluster command. Chimeric sequences were screened from the sequences by chimera.vsearch with default settings and the remaining sequences taxonomically classified (classify.seqs) to the SILVA 132 taxonomy reference. Sequences were assigned to operational taxonomic units (OTUs) by cluster.split command with the cutoff set at 0.03 (97% identity). Alpha diversity was calculated by Shannon's diversity index, Simpson's diversity index, and observed species richness (OSR); beta diversity was determined by calculating Bray–Curtis dissimilarity index and visualized as an NMDS plot.

### 2.7 Statistical analysis

A McDonald–Kreitman test (http://mkt.uab.es/mkt/mkt.asp) was performed for US populations (CNM, NB, BC) against Mexican populations (RS, AH) for both *rbcL* and *matK* gene sequences to determine divergence between the US and MX populations. Sequence alignments generated in Clustal Omega were used to calculate dS (synonymous substitution) and dN (nonsynonymous substitution) across all samples and between US and MX Shaw's Agave samples per locus in SNAP v2.1.1. pH and moisture content variation per site were analyzed *via* one‐way ANOVA with post hoc Bonferroni correction for multiple comparison testing at *p* < 0.001. *t*‐tests assuming unequal variances were performed to compare richness (S) and evenness (E) of microbial activity between sites. AMOVA (analysis of molecular variance) and HOMOVA (homogeneity of molecular variance) were calculated between different geographic clusters of Shaw's Agave for barcoding and 16S rRNA data. Pearson's linear correlation coefficients were generated between all soil‐related indices and measurements.

## 3 RESULTS

### 3.1 Phylogenetics

**FIGURE 2:**
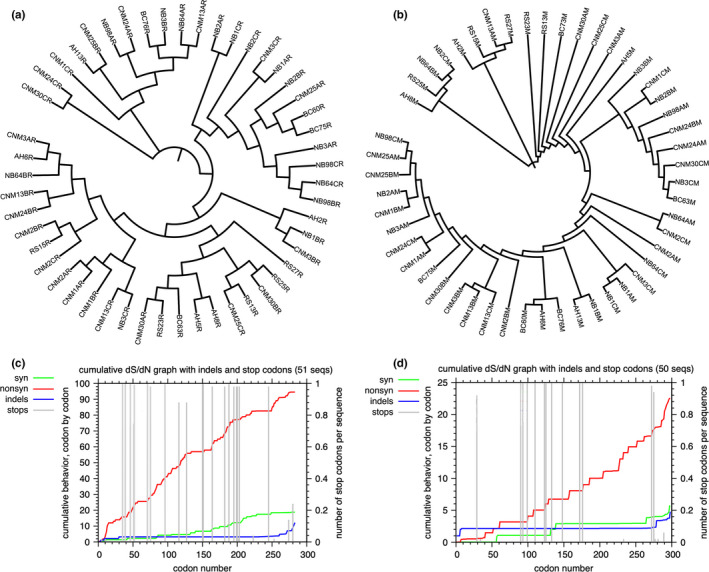
Unrooted phylogenetic tree of Shaw's Agave generated in iTOL for *rbcL* and *matK* sequences. Sample names consist of geographic source (e.g., "CNM" or "NB"), plant identifier, and biological replicate (e.g., A/B/C). CNM = Cabrillo National Monument, NB = Point Loma Naval Base, BC = Border Fields, RS = Rosarito, and AH = Arroyo Hondo. (a) The suffix "R" in the above figure refers to the gene *rbcL,* while (b) the suffix "M" in the above figure refers to the gene *matK*. Phylogenetic trees were generated in Clustal Omega using neighbor‐joining and further edited in the Interactive Tree of Life (iTOL). Radial bars represent evolutionary time distances from common ancestors. Synonymous–nonsynonymous analysis of (c) *rbcL* and (d) *matK* was performed for all sample pairs using SNAP v2.1.1.

Seventeen neutral polymorphisms and 65 non‐neutral polymorphisms were identified between the US and MX sites for the *rbcL* locus. One neutral polymorphism and 9 non‐neutral polymorphisms were detected between the US and MX sites for the *matK* locus; no divergences were detected for either locus. Synonymous/nonsynonymous analysis shows relatively equal distribution of both synonymous and nonsynonymous variations across the two barcoding loci (Figure 2c,d). Overall, averaged pairwise comparisons across all samples for the *rbcL* gene showed dS = 0.013387, dN = 0.013016, and dS/dN = 1.028503; for the *matK* gene, averages of all pairwise comparisons showed dS = 0.005579, dN = 0.007400, and dS/dN = 0.753919. Comparing US agave samples and MX agave samples, the *rbcL* gene has dS = 0.0142, dN = 0.0116, and dS/dN = 1.5070, while the *matK* gene has dS = 0.0042, dN = 0.0035, and dS/dN = 1.4176.

Genetic variances among the five populations (CNM, NB, BC, RS, and AH) are statistically significant for the *rbcL* locus but not for the matK locus (*p* < 0.001, AMOVA, Table S1). The following AMOVA pairwise comparisons for the rbcL locus are significantly different: 1. AH to BC (*p* = 0.013), 2. AH to RS (*p* = 0.016), 3. BC to CNM (*p* = 0.008), 4. BC to NB (*p* = 0.038), and 5. BC to RS (*p* = 0.037; AMOVA, Table S1). For the *matK* locus, the only population pair that was significantly different was BC to RS (*p* = 0.013; Table S1). For *rbcL*, the following pairwise comparisons are significantly different: 1. AH to BC (*p* = 0.037), 2. AH to NB (*p* < 0.001), 3. BC to NB (*p* = 0.026), 4. BSFP to RS (*p* = 0.044), and 5. NB to RS (*p* < 0.001; HAMOVA, Table S2). For *matK*, the following pairwise comparisons were found to be significantly different: 1. BC to RS (*p* = 0.011), 2. CNM to RS (*p* = 0.001), and 3. NB to RS (*p* = 0.035; HAMOVA, Table S2). Genetic similarities between samples of different geographic sites for the *rbcL* and *matK* loci can be visualized in Figure 2.

### 3.2 Shaw's Agave soil properties differ by location

**FIGURE 3:**
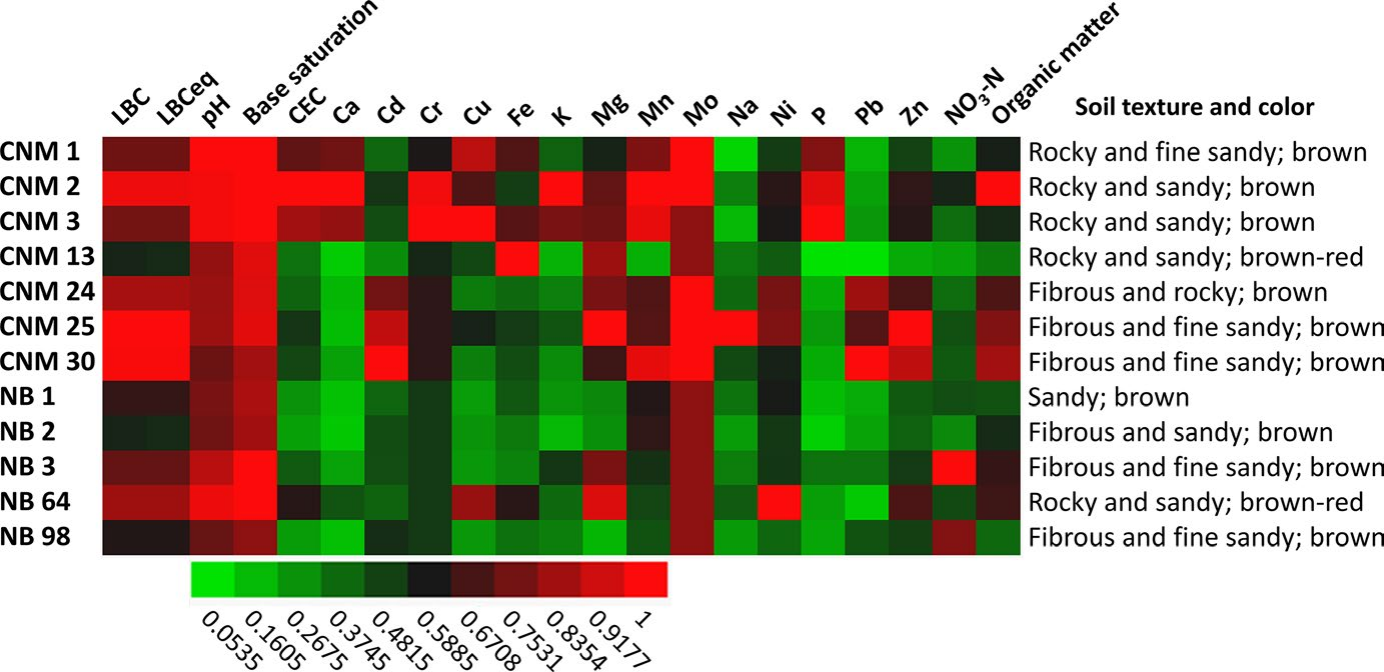
Characteristics of Point Loma agave soil samples. Rows represent sample sites at Point Loma, and columns represent characteristics of soil. CNM = Cabrillo National Monument and NB = Point Loma Naval Base. Numbers (1–98) represent *Agave shawii* ssp. *shawii* individuals. Measurements of each chemical property (available in Table S4) were normalized to the highest value for that property and plotted. Green indicates relatively lower values, and red indicates relatively higher values.

While soil samples within Point Loma varied in moisture content, soil from NB had less overall moisture (avg = 1.36%) than CNM samples (avg = 4.73%; *p* < 0.0001; Table S3). pH values of pooled soil samples were within one standard deviation of the corresponding mean values of individual pH measurements of each replicate (Table S3, Table S4). NB samples were more acidic pH (avg = 6.54) than CNM samples (avg = 7.12; *p* < 0.05; Table S3). pH values clustered closely together for all samples except those from CNM 25 (SD = 0.92) and CNM 30 (SD = 1.34). pH was significantly and positively correlated to moisture percentage (*p* < 0.05; *ρ* = 0.4173), and evenness of microbial activity (*p* < 0.05; *ρ* = 0.4622). Figure 3 summarizes soil chemical properties and visual characterization. Chemical measurements of pooled soil samples are shown in Table S4.

**FIGURE 4:**
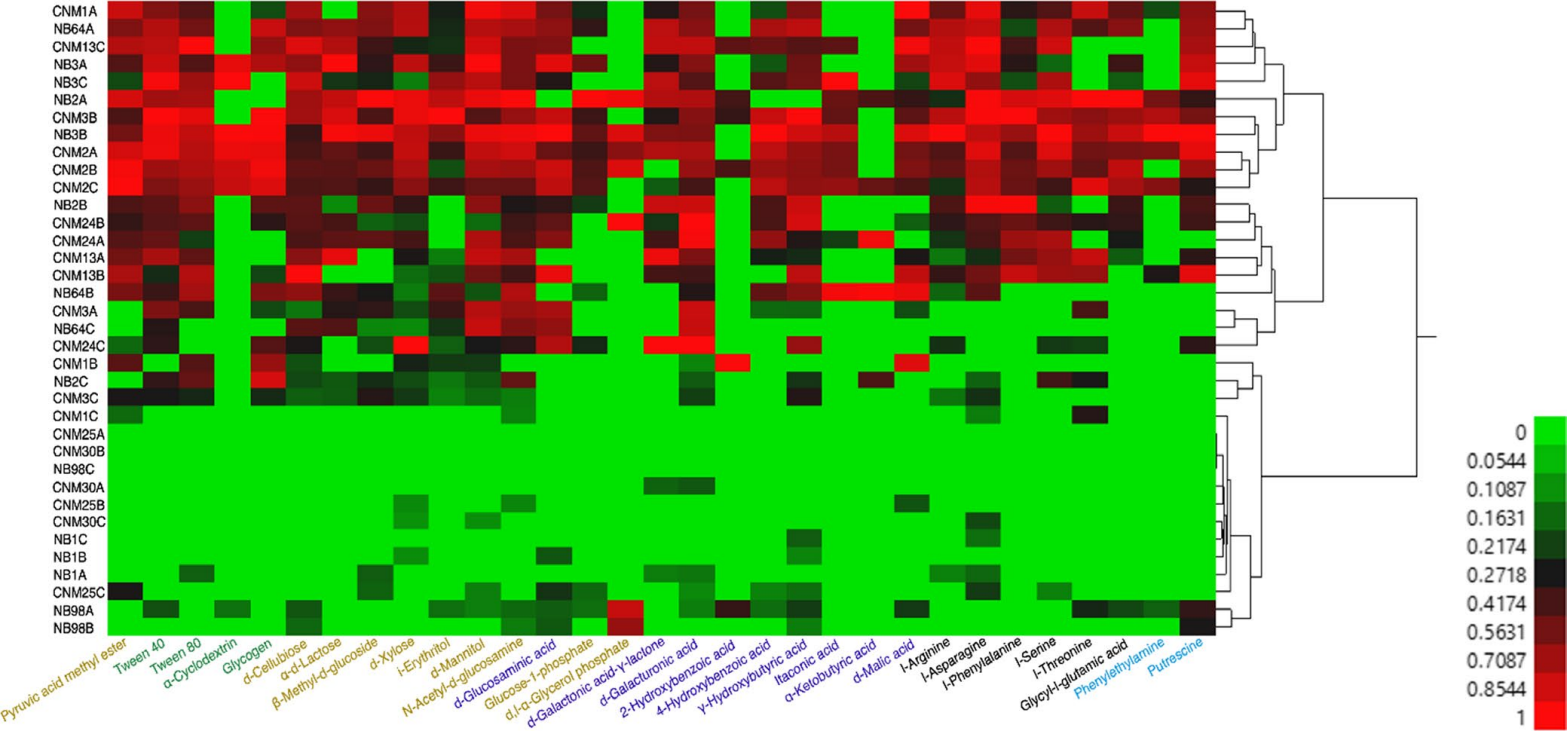
Hierarchical clustering of microbial carbon source utilization profiles by sampling site. Rows represent single sampling events in Point Loma, and columns represent individual carbon sources. CNM = Cabrillo National Monument and NB = Point Loma Naval Base. Numbers (1–98) represent *Agave shawii* ssp. *shawii* individuals and A/B/C are biological replicates. Heatmap colors (green‐red) represent normalized absorbance values postincubation normalized to preincubation absorbances and to the control well. Hierarchical clustering was generated in JMP Pro and Graphic. Carbon sources are labeled yellow (carbohydrates), green (polymers), purple (carboxylic and ketonic acids), black (amino acids), and blue (amine/amides).

### 3.3 Shaw's Agave soil microbes' carbon source utilization varies greatly

Microbial metabolism assay analysis revealed differences in richness across Shaw's Agave individuals and relatively even distributions of utilized sources. Shannon's evenness index near 0.7 was characteristic for CNM 25, CNM 30, and NB 98 samples, clustering within a major “low richness” group (Table 1, Figure 4). Soil from the same agave plant, while exhibiting a similar metabolic profile, varied in microbial community structure. CNM 1, CNM 3, and NB 64 samples varied significantly between replicates in richness (Table 1). The remaining soil samples were not significantly different in richness and evenness (Table 1, Figure 4). Soil microbes from CNM 25, CNM 30, and NB 1 had the lowest average richness with 4, 2, and 4 carbon sources utilized, respectively. D‐galacturonic acid, n‐acetyl‐d‐glucosamine, d‐mannitol, d‐xylose, d‐cellobiose, and γ‐amino butyric acid are the most commonly utilized carbon sources on the EcoPlate, with over 24 samples (out of 36) having biologically relevant (Garland, 1997) color changes.

**TABLE 1:** Functional biodiversity of Point Loma agave soil microbiomes

Site	Shannon's Evenness (SD)	Shannon's Richness (SD)	Shannon's Index (SD)
CNM 1	0.97 (0.02)	11.33 (8.08)	2.22 (0.86)
CNM 2	0.98 (0.00)	28.67 (0.58)	3.28 (0.02)
CNM 3	0.96 (0.03)	19.67 (7.23)	2.83 (0.35)
CNM 13	0.96 (0.01)	22.0 (1.73)	2.96 (0.11)
CNM 24	0.95 (0.03)	21.0 (3.0)	2.88 (0.21)
CNM 25	0.66 (0.57)	4.33 (5.13)	1.12 (1.14)
CNM 30	0.65 (0.57)	1.67 (1.53)	0.58 (0.54)
NB 1	0.99 (0.00)	3.67 (2.08)	1.19 (0.55)
NB 2	0.96 (0.1)	21.33 (5.03)	2.89 (0.25)
NB 3	0.97 (0.01)	23.67 (5.03)	3.15 (0.15)
NB 64	0.96 (0.03)	18.33 (7.64)	2.72 (0.52)
NB 98	0.66 (0.57)	8.0 (9.17)	1.46 (1.44)

Note

EcoPlates containing a suspension of soil microbes were incubated for 7 days and tested for color development. Wells with an absorbance change larger than 0.25 were considered a positive test. Mean values of Shannon's evenness, richness, and index were calculated from the 3 biological replicates of soil per site (*n* = 3). Numbers (1–98) represent *Agave shawii* ssp. *shawii* individuals. Sites CNM 25A, CNM 30B, NB 98C had scores of 0 across evenness, richness, and Shannon's index. From CNM, evenness ranged from 0 to 0.995, richness ranged from 0 to 28, and Shannon's index ranged from 0 to 3.24. From NB, evenness ranged from 0 to 0.997, richness ranged from 0 to 29, and Shannon's index ranged from 0 to 3.31. There is no statistically significant difference between the two sites for any of the three diversity indicators.

### 3.4 16S rRNA microbial diversity

**FIGURE 5:**
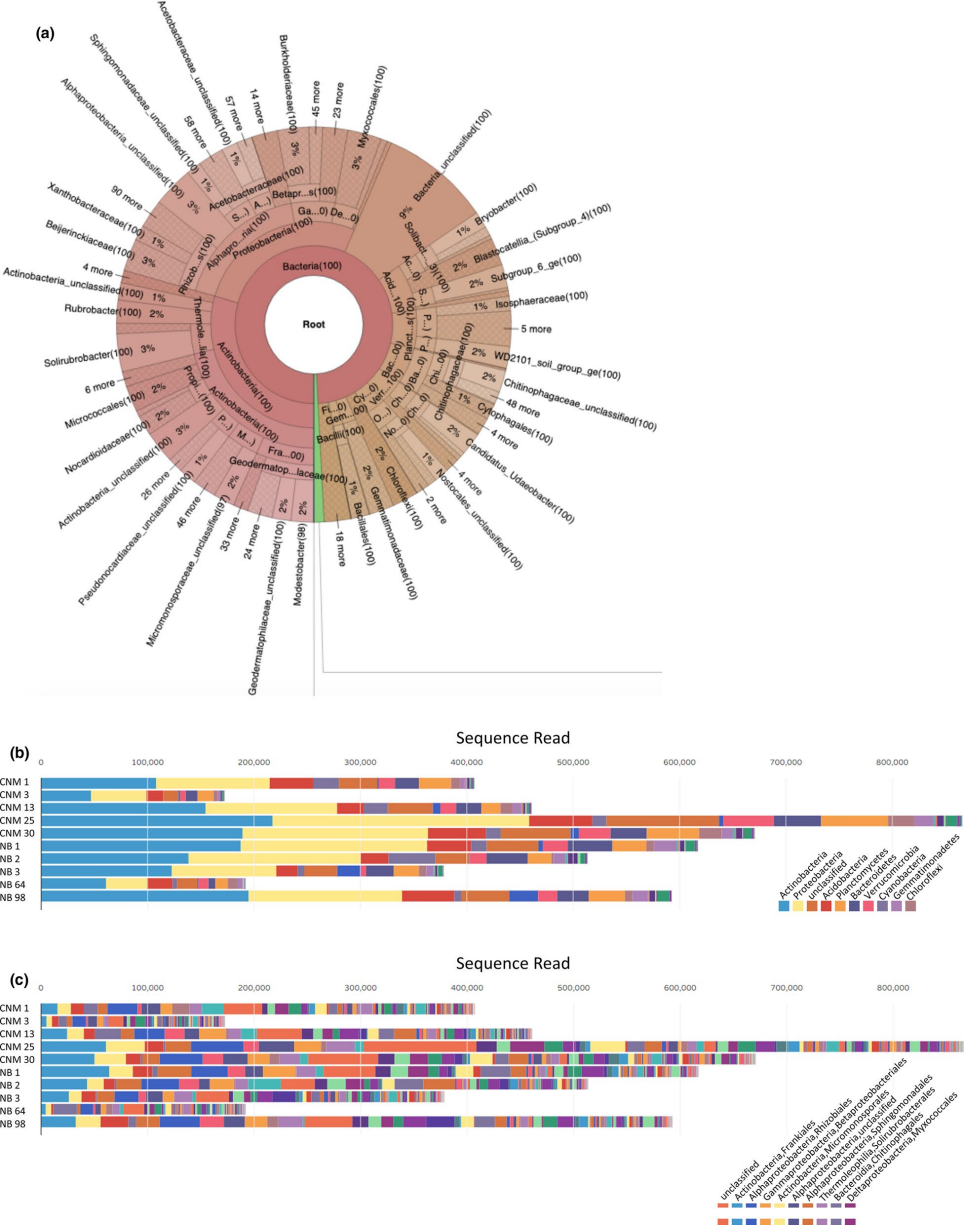
Identified soil microbial species across Point Loma sites. A) Krona pie chart showing microbial taxon proportions from 16S rRNA sequences collected across all Point Loma sites (CNM and NB). An interactive version is linked: https://usegalaxy.org/datasets/bbd44e69cb8906b5831a2421e90be777/display/?preview=True&dataset=0&node=0&collapse=true&color=false&depth=6&font=11&key=true, which displays all identified microbial species data on lower taxonomic ranks. B) Bar chart showing the distribution of microbial phyla for each sampling site. The legend shows the top ten enriched phyla. C) Bar chart showing the distribution of microbial orders for each sampling site. The legend shows the top ten enriched orders.

**FIGURE 6:**
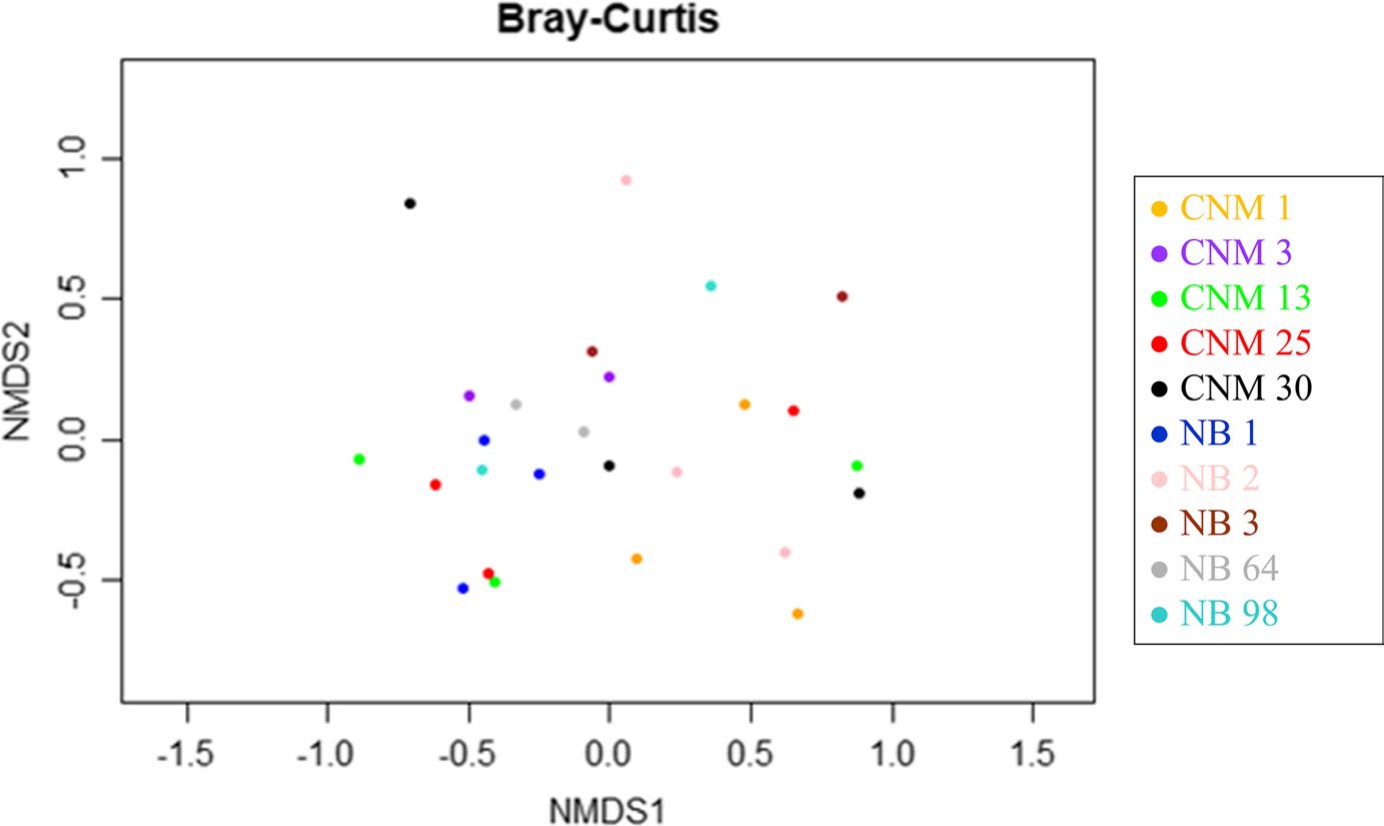
Bray–Curtis dissimilarity NMDS plot between Cabrillo National Monument (CNM) and Point Loma Naval Base (NB) of agave sample sites based on 16S operational taxonomic unit (OTU) clustering: Beta diversity calculated by Bray–Curtis dissimilarity calculator between and among all agave sample sites on the California coast by microbial communities clustered in OTUs, with minimum confidence 97%. Numbers (1–98) represent *Agave shawii* ssp. *shawii* individuals. There is no significant clustering for each site.

99% of soil microbial species identified are bacteria, 0.02% are Archaea, and 0.8% are unclassified. Phylum Actinobacteria comprises 30% of classified bacterial, followed by Proteobacteria (27%), unclassified bacteria (9%), Acidobacteria (7%), and Bacteriodetes and Planctomycetes (6% each). Bacterial species <5% include Verrucomicrobiae, Cyanobacteria, Gemmatimonadetes, Chloroflexi, and Firmicutes (Figure 5). Alpha diversity calculators reveal similarities within CNM and NB site locations; at an OTU clustering threshold of 97%, Shannon's calculated diversity indices range from 6.1832 to 6.7691 across all samples, and Simpson's calculated diversity indices range from 0.0040 to 0.0079 (Table S5). The Bray–Curtis beta diversity calculator suggests no specific microbial community structure distinguishes CNM from NB samples (Figure 6).

### 3.5 Correlation between soil properties, carbon utilization, and 16S rRNA diversity

Base saturation is significantly correlated with Shannon's Index of soil microbe functional diversity (*p* < 0.05, *r* ^2^ = 0.3676). Lime buffer capacity (*p* < 0.01, *r* ^2^ = 0.7055), equilibrium lime buffer capacity (*p* < 0.01, *r* ^2^ = 0.7142), and zinc content of the soil (*p* < 0.01, *r* ^2^ = 0.7136) are significantly correlated with Shannon's Index of soil microbe structural diversity. There is no correlation between 16S rRNA diversity and carbon source utilization or functional diversity.

## 4 DISCUSSION

We utilized *rbcL* and *matK* barcoding genes, microbial enzymatic assays, and 16S rRNA sequencing to assess the genetic diversity of Shaw's Agave and determine the plants’ microbiome and its functional diversity. This rare succulent species is not clonal and exhibit genetic diversity across its habitat range. Low enzymatic activity in 36% of *Agave* soils samples may be indicative of compromised fitness among transplanted plants. 16S rRNA analysis suggests that *Agave*’s microbiota is diverse within and between sites.

### 4.1 Phylogenetics

Sequenced *rbcL* and *matK* loci indicate similar genetic diversity between populations on the Point Loma peninsula, the Border Fields, and northern Baja California, Mexico (Figure 2). There is no obvious clustering across samples from the same geographic site, suggesting a greater than expected level of genetic diversity among Shaw's Agave at Point Loma. Moreover, the genetic diversity between the rosettes within a cluster indicates that either Shaw's Agave in Point Loma are not all clonally propagated, or that transplanted individuals were from different locations, but there are no documents to verify this. The diversity between rosettes suggests sexual propagation and potential for diversification of the population’s gene pool. Surprisingly, pairwise comparisons across Shaw's Agave populations showed more significant differences in *rbcL* than in *matK*. Nevertheless, the overall higher dS/dN of *rbcL* (1.028503) compared to *matK* (0.753919) suggests that *rbcL* has relatively higher rates of synonymous substitutions, meeting expectation that *rbcL* is relatively more conserved. Considering the functional relevance of substitutions, higher nonsynonymous mutation rates observed in *matK* resulting in amino acid changes might be more structurally or functionally consequential. Comparing the US and MX Shaw's Agave population, the *rbcL* locus has dS/dN = 1.5070, and the *matK* locus has dS/dN = 1.4176. The relatively higher amount of synonymous variations suggest that most of the differences in the two loci between sampled US and MX agave plants are neutral, further illustrating substantial similarity between the US and MX Shaw's Agave population. Our data show that the US Shaw's Agave population is not clonal, and its genetic diversity within the two barcoding regions is comparable with that of the healthy MX Shaw's Agave population.

The population of Shaw's Agave was likely continuous across its range before intense coastal urban development and a new border wall construction fragmented and isolated the US population of Shaw's Agave (Figure 1g). Genetic similarities between California and northern Baja California populations are still evident despite this fragmentation. We speculate that recent urban development and the border wall construction have compromised the genetic diversity of local Shaw's Agave, but the species' long reproductive cycle masks the evidence of significant divergence between populations.

### 4.2 Differences in soil between Cabrillo National Monument and Naval Base Point Loma

CNM soil is significantly more alkaline (*p* < 0.05) and moist (*p* < 0.0001) than NB, and CNM soil microbes use significantly more carbon sources then NB soil microbes (*p* < 0.05). Although both sites are within Point Loma Peninsula, differences in altitude, soil type, and proximity to the ocean may contribute to such variation. The higher pH and moisture of CNM may be a more suitable environment for microbial metabolic carbon utility (Bell et al., 2008; Zhalnina et al., 2014). Our data show no indication of heavy metal contamination in the Point Loma Shaw's Agave habitat (Table S4).

Soil microbial metabolic activity assay shows large variations both within and between sites. In the metabolic profiling of CNM and NB soil, three major clusters arise: two metabolically active and one relatively inactive agave microbiota enzyme activity clusters. Low metabolic activity samples (CNM 25 A/B/C, CNM 30 A/B/C, NB 1 A/B/C, NB 98 A/B/C) show low variability between replicates, which correlates with reduced evenness due to the limited carbon metabolism. Soil from two major sampling locations (CNM and NB) is distributed evenly among all clusters, suggesting associated microbial activities are individual‐specific rather than location‐specific. Such a pattern could be the result of agave replantation efforts in the past. If so, the current microbiome is likely be the result of interactions between the agave plant original holobiome at the time of replantation and existing soil microbiome of the replantation site environment. Our results imply that each microbiome reflects individual‐specific holobiome adaptation to a new environment. Agave‐associated soil microbes displaying little to no enzymatic activity may be indicative of more recent agave transplants. The most commonly used carbon sources (d‐galacturonic acid, n‐acetyl‐d‐glucosamine, d‐mannitol, d‐cellobiose, and γ‐amino butyric acid) are typical for soil microbiome (Almendras et al., 2018; Gomez et al., 2006; Teng et al., 2010). Base saturation, the percentage of base cations (Ca, Mg, K, etc.) among total cations, is significantly positively correlated with functional diversity (*p* < 0.05, *r* ^2^ = 0.3676). Calcium and magnesium amendments have been shown to increase soil microbial diversity at soil horizon A1, which is within our sampling depth range (Balland‐Bolou‐Bi & Poszwa, 2012) suggesting base saturation as an important marker of soil microbial activity in the Point Loma Shaw's Agave habitat. The association between the plant and its microbiota is a critical aspect of plant fitness (Rodriguez & Durán, 2020; Rosenberg et al., 2010; Trivedi et al., 2020). Our results reveal that, following the replantation event, each plant and associated microbiota could have experienced individual‐specific adaptation processes, which is dependent on both the holobiome origin and characteristics of the new environment, such as the soil quality and local microorganisms.

### 4.3 16S rRNA microbial diversity

16S rRNA sequencing revealed that the majority of pooled agave microbiota consists of proteobacteria and actinobacteria (Figure 5). Of the Proteobacteria, the majority are nitrogen‐fixing Alphaproteobacteria, Gammaproteobacteria, and Deltaproteobacteria. Of the Actinobacteria, most are Micromonosporaceae, critical for organic plant matter degradation. Rarefaction curves based on OTUs show similar sampling coverage (Figure S1), while beta diversity compared across Bray–Curtis indices suggest no trend in microbial species composition nor consistent microbial predictor between two sampling sites 5 km apart. Dissimilarity in soil microbe profile is not only limited to geographically distant sampling sites, but also within compact local agave clusters (Figure 6, Table S6). Lime buffer capacity (*p* < 0.01, *r* ^2^ = 0.7055) and equilibrium lime buffer capacity (*p* < 0.01, *r* ^2^ = 0.7142) are significantly positively correlated with 16S rRNA structural diversity of soil microbe. As microbe needs to adapt to pH changes (Bååth, 1996; Sheng & Zhu, 2018), soil resisting significant changes in pH should promote the establishment of a stable and possibly more diverse microbial community. Zinc at trace levels (1.99~9.88 mg/kg) is also positively correlated (*p* < 0.01, *r* ^2^ = 0.7136) with the structural diversity of soil microbe in the Point Loma Shaw's Agave habitat. Zinc as a micronutrient is essential for bacterial for structural and enzymatic functions (Andreini et al., 2006; Gielda & Diritaa, 2012). The positive correlation suggests that maintaining the higher detected level of zinc (6.82–9.88 mg/kg) is beneficial for the microbial diversity in this habitat. Shannon's calculated diversity indices range from 6.1832 to 6.7691 across all samples, and Simpson's calculated diversity indices range from 0.0040 to 0.0079 (Table S5, Table S7). From the calculated Shannon's diversity indices, there are e^6.1832^ (about 485) to e^6.7691^ (about 871) equally common species. Although Simpson's diversity indices indicate a relatively low diversity of the microbial community, Shannon's calculated diversity indices show this level of diversity is normal for agaves (Coleman‐Derr et al., 2016). Due to conservation concerns, we did not sample Shaw's Agaves' rhizosphere directly, in which the soil is directly adjacent to the roots, to avoid disrupting their shallow and delicate root system (direct communication with NPS research and management staff). We made efforts to stay within the reasonable proximity of the peripheral root system (~5 cm). Admittedly, the rhizosphere has higher microbial variation than the bulk soil, and should yield more direct data regarding the association between plant health and soil microbiome (Coleman‐Derr et al., 2016). As an initial representative survey of the microbial community makeup for these vulnerable US agave populations, future microbial analysis can be compared and potentially highlight community trends.

## 5 CONCLUSION

Sequences at the two barcoding loci, *rbcL* and *matK*, show similar genetic variation among five Shaw's agave populations in United States and Mexico. Soil microbiome analysis within the CNM population reveals large individual variations and relatively low but overall expected diversity for *Agave* species. Our results show close relationship between the US and MX agave populations and demonstrate the lack of agave sprouts at Point Loma is unlikely caused by the lack of genetic or soil microbial diversity. Further efforts are needed to examine other fitness factors that might have impeded Shaw's Agave's reproductive success.

## 6 LIMITATIONS

Phylogenetic analysis of the barcoding gene regions *rbcL* and *matK* is a prequel for deeper sequencing of more comprehensive hypervariable loci, such as *ycf1*. Both *rbcL* and *matK* genes encoded in chloroplast are standard DNA barcodes for land plants (Hollingsworth et al., 2009). While *rbcL* is highly conserved and allows for differentiation between many species, it does not have great discrimination power within species; *rbcL* performs well in combination with other loci (Hollingsworth et al., 2009). For instance, *matK* has high levels of discrimination power between angiosperm species and is often used in combination with *rbcL* (Burgess et al., 2011; Hollingsworth et al., 2009). The *matK*/*rbcL* combination is the standard two‐locus DNA barcode (Li et al., 2011). While using multiple loci would increase resolution and power, a 2‐locus barcode was chosen for cost‐effectiveness (Burgess et al., 2011; Hollingsworth et al., 2009). However, sequencing more expansive loci will yield a statistically more powerful phylogenetic analysis. The barcoding method is also relatively limited in its discrimination resolution when differentiating between individuals within a species, while more powerful genetic markers such as SSRs and SNPs will yield more accurate and conclusive genetic diversity data.

Low sample size and lack of robust morphological markers among geographically distant agave, while do not affect the testing of our hypothesis, limit further site‐specific gene–environment interaction analysis and detection of speciation evidence. The agave sampling was limited by a combination of budget constraints, the number of previously collected individual plants from Mexico, and restricted public access to plants within USA sites; 12 plants sampled at the CNM and NB were chosen by the NPS due to their locations at a relatively high visitor activity areas throughout the park.

Soils were sampled during a single dry‐season event, and temporal variation was not assessed. Soil type (sandy, high in organic matter) may influence the quality of DNA as high levels of organic matter can interfere with extraction protocols (pers. comm. Zymo Research). The microorganisms in the rhizosphere would reflect the plants' health more accurately than those in the bulk soil (Korenblum et al., 2020). The challenge of metabolic profiling is the selection of culturable microorganisms under laboratory conditions. Such a method favors fast‐growing microbes and reflects potential rather than in situ metabolic diversity. Contribution of certain microbes may be over‐ or underrepresented on Biolog EcoPlates; carbon sources provided by the EcoPlate are not representative of those found in the soil. Microbial diversity analysis via 16S rRNA sequencing needs revisions pending database updates, while little is known about the spatial and temporal variability of soil microbes.

## 7 FUTURE DIRECTIONS

This study offers insights about Shaw's Agave population dynamics that may affect the health of the species. The Shaw's Agave populations in Point Loma (CNM, NB) exhibit similar levels of genetic variation to populations in northern Baja California populations in the *rbcL* and *matK* loci. To draw further conclusions on population clonality, the asexual genetic variability of Shaw's Agave should be determined. Within the United States, Shaw's Agave's long‐term sustainability is complex and vulnerable, confounded by long reproductive cycles, herbivory, habitat loss, and/or low fecundity. Preliminary studies indicate that herbivory may be severely impacting the germination of new individuals in the wild (pers. comm. K. Lombardo, 2020). Continued monitoring would help formulate mitigation strategies promoting Shaw's Agave conservation. While our study examines the relative gene diversity and soil microbiome diversity, determining why this species provides so few viable seeds and quantifying fecundity and long‐term monitoring of these aspects of fitness are recommended. Future studies may investigate the *Agave* natural pollination by the Mexican long‐tongued bat or the effectiveness of anthropogenically induced pollination. Correlating Agave‐associated soil moisture and pH in the US and northern Baja California could guide reintroduction and propagation efforts. Finally, the microbiota was identified to the taxonomic order or genus level and these microbes cannot be identified as known pathogenic or symbiotic bacteria. Pathogenicity tests could determine if CNM and NB agave populations are diseased. To ensure the long‐term survival and fitness of Shaw's Agave, continued ecological risk assessment including genetic diversity is recommended.

## CONFLICT OF INTEREST

The authors declare that they have no conflict of interest.

## AUTHOR CONTRIBUTIONS

**Jeanne P. Vu:** Data curation (lead); formal analysis (lead); investigation (equal); methodology (equal); project administration (equal); visualization (lead); writing – original draft (equal); writing – review and editing (equal). **Miguel F. Vasquez:** Data curation (equal); formal analysis (equal); methodology (supporting); validation (equal); visualization (equal); writing – review and editing (supporting). **Zuying Feng:** Data curation (supporting); formal analysis (supporting); validation (equal); writing – original draft (supporting); writing – review and editing (equal). **Keith Lombardo:** Conceptualization (lead); funding acquisition (lead); investigation (equal); project administration (equal); resources (lead); supervision (equal); writing – review and editing (equal). **Sora Haagensen:** Data curation (supporting); formal analysis (supporting); investigation (supporting); visualization (supporting); writing – review and editing (supporting). **Goran Bozinovic:** Conceptualization (lead); data curation (supporting); formal analysis (equal); funding acquisition (lead); investigation (lead); methodology (lead); project administration (lead); resources (equal); supervision (lead); validation (lead); visualization (equal); writing – original draft (lead); writing – review and editing (lead).

## Supporting information

Figure S1

Table S1

Table S1.1

Table S2

Table S3

Table S4

Table S5

Table S6

Table S7.1

Table S7.2

Table S7.3

Table S7.4

Table S7.5

Table S7.6

Table S7.7

Table S7.8

Table S7.9

Supplementary Material

## Data Availability

DNA sequences: GenBank accession MT679257–MT679307, MT679308–MT679357. 16S rRNA sequences: GenBank accession KEEQ00000000. Dryad accession https://doi.org/10.6076/D1C884.

## References

[ece37172-bib-0001] Afgan, E. , Baker, D. , Batut, B. , Van Den Beek, M. , Bouvier, D. , Ech, M. , Chilton, J. , Clements, D. , Coraor, N. , Grüning, B. A. , Guerler, A. , Hillman‐Jackson, J. , Hiltemann, S. , Jalili, V. , Rasche, H. , Soranzo, N. , Goecks, J. , Taylor, J. , Nekrutenko, A. , & Blankenberg, D. (2018). The Galaxy platform for accessible, reproducible and collaborative biomedical analyses: 2018 update. Nucleic Acids Research, 46(W1), W537–W544. 10.1093/nar/gky379 29790989 PMC6030816

[ece37172-bib-0002] Almendras, K. , Leiva, D. , Carú, M. , & Orlando, J. (2018). Carbon consumption patterns of microbial communities associated with Peltigera lichens from a Chilean temperate forest. Molecules, 23(11), 2746 10.3390/molecules23112746 PMC6278465 30355963

[ece37172-bib-0003] Andreini, C. , Banci, L. , Bertini, I. , & Rosato, A. (2006). Zinc through the three domains of life. Journal of Proteome Research, 5(11), 3173–3178. 10.1021/pr0603699 17081069

[ece37172-bib-0004] Bååth, E. (1996). Adaptation of soil bacterial communities to prevailing pH in different soils. FEMS Microbiology Ecology, 19(4), 227–237. 10.1016/0168-6496(96)00008-6

[ece37172-bib-0005] Balland‐Bolou‐Bi, C. , & Poszwa, A. (2012). Effect of calco‐magnesian amendment on the mineral weathering abilities of bacterial communities in acidic and silicate‐rich soils. Soil Biology and Biochemistry, 50, 108–117. 10.1016/j.soilbio.2012.02.034

[ece37172-bib-0006] Bartlett, L. J. , Newbold, T. , Purves, D. W. , Tittensor, D. P. , & Harfoot, M. B. J. (2016). Synergistic impacts of habitat loss and fragmentation on model ecosystems. Proceedings of the Royal Society B: Biological Sciences, 283(1839), 20161027 10.1098/rspb.2016.1027 PMC5046893 27655763

[ece37172-bib-0007] Beauchamp, R. M. (1976). Moving day for Agave. Fremontia, 4(1), 22–23.

[ece37172-bib-0008] Bell, C. , McIntyre, N. , Cox, S. , Tissue, D. , & Zak, J. (2008). Soil microbial responses to temporal variations of moisture and temperature in a Chihuahuan Desert grassland. Microbial Ecology, 56(1), 153–167. 10.1007/s00248-007-9333-z 18246293

[ece37172-bib-0009] Browne, L. , & Karubian, J. (2018). Habitat loss and fragmentation reduce effective gene flow by disrupting seed dispersal in a neotropical palm. Molecular Ecology, 27(15), 3055–3069. 10.1111/mec.14765 29900620

[ece37172-bib-0010] Burgess, K. S. , Fazekas, A. J. , Kesanakurti, P. R. , Graham, S. W. , Husband, B. C. , Newmaster, S. G. , Percy, D. M. , Hajibabaei, M. , & Barrett, S. C. H. (2011). Discriminating plant species in a local temperate flora using the rbcL+matK DNA barcode. Methods in Ecology and Evolution, 2(4), 333–340. 10.1111/j.2041-210X.2011.00092.x

[ece37172-bib-0011] Cagnolo, L. , Valladares, G. , Salvo, A. , Cabido, M. , & Zak, M. (2009). Habitat fragmentation and species loss across three interacting trophic levels: Effects of life‐history and food‐web traits. Conservation Biology, 23(5), 1167–1175. 10.1111/j.1523-1739.2009.01214.x 19765035

[ece37172-bib-0012] California Native Plant Society (2020). Inventory of rare and endangered plants. California Native Plant Society.

[ece37172-bib-0013] Coleman‐Derr, D. , Desgarennes, D. , Fonseca‐Garcia, C. , Gross, S. , Clingenpeel, S. , Woyke, T. , North, G. , Visel, A. , Partida‐Martinez, L. P. , & Tringe, S. G. (2016). Plant compartment and biogeography affect microbiome composition in cultivated and native Agave species. New Phytologist, 209(2), 798–811. 10.1111/nph.13697 PMC5057366 26467257

[ece37172-bib-0014] Frankham, R. , Ballou, J. D. , & Briscoe, D. A. (2002). Loss of genetic diversity in small populations In Introduction to conservation genetics. (pp. 227–253). Cambridge University Press 10.1017/cbo9780511808999

[ece37172-bib-0015] Garland, J. L. (1997). Analysis and interpretation of community‐level physiological profiles in microbial ecology. FEMS Microbiology Ecology, 24(4), 289–300. 10.1016/S0168-6496(97)00061-5

[ece37172-bib-0016] Gentry, H. S. (1978). The agaves of Baja California. Occasional Papers of the California Academy of Sciences, 130, 1–119.

[ece37172-bib-0017] Gielda, L. M. , & Diritaa, V. J. (2012). Zinc competition among the intestinal microbiota. Mbio, 3(4), e00171‐12 10.1128/mBio.00171-12 22851657 PMC3419517

[ece37172-bib-0018] Gomez, E. , Ferreras, L. , & Toresani, S. (2006). Soil bacterial functional diversity as influenced by organic amendment application. Bioresource Technology, 97(13), 1484–1489. 10.1016/j.biortech.2005.06.021 16168637

[ece37172-bib-0019] Grover, M. , Ali, S. Z. , Sandhya, V. , Rasul, A. , & Venkateswarlu, B. (2011). Role of microorganisms in adaptation of agriculture crops to abiotic stresses. World Journal of Microbiology and Biotechnology, 27(5), 1231–1240. 10.1007/s11274-010-0572-7

[ece37172-bib-0020] Gryta, A. , Frąc, M. , & Oszust, K. (2014). The application of the Biolog EcoPlate approach in ecotoxicological evaluation of dairy sewage sludge. Applied Biochemistry and Biotechnology, 174(4), 1434–1443. 10.1007/s12010-014-1131-8 25119549 PMC4177563

[ece37172-bib-0021] Hollingsworth, P. M. , Forrest, L. L. , Spouge, J. L. , Hajibabaei, M. , Ratnasingham, S. , van der Bank, M. , Chase, M. W. , Cowan, R. S. , Erickson, D. L. , Fazekas, A. J. , Graham, S. W. , James, K. E. , Kim, K. J. , John Kress, W. , Schneider, H. , van AlphenStahl, J. , Barrett, S. C. H. , van den Berg, C. , & Bogarin, D. , … Little, D. P. (2009). A DNA barcode for land plants. Proceedings of the National Academy of Sciences of the United States of America, 106(31), 12794–12797. 10.1073/pnas.0905845106 19666622 PMC2722355

[ece37172-bib-0022] Holt, R. D. , & Loreau, M. (2002). Biodiversity and ecosystem functioning: The role of trophic interactions and the importance of system openness In The functional consequences of biodiversity. (pp. 246–262). Princeton University Press 10.1515/9781400847303.246

[ece37172-bib-0023] Keller, L. F. , & Waller, D. M. (2002). Inbreeding effects in wild populations. Trends in Ecology and Evolution, 17(5), 230–241. 10.1016/S0169-5347(02)02489-8

[ece37172-bib-0024] Kissel, D. E. , & Vendrell, P. F. (2012). Soil testing: Soil pH and salt concentration, (Circular 875, pp. 1–2). The University of Georgia Cooperative Extension.

[ece37172-bib-0025] Korenblum, E. , Dong, Y. , Szymanski, J. , Panda, S. , Jozwiak, A. , Massalha, H. , Meir, S. , Rogachev, I. , & Aharoni, A. (2020). Rhizosphere microbiome mediates systemic root metabolite exudation by root‐to‐root signaling. Proceedings of the National Academy of Sciences of the United States of America, 117(7), 3874–3883. 10.1073/pnas.1912130117 32015118 PMC7035606

[ece37172-bib-0026] Kozich, J. J. , Westcott, S. L. , Baxter, N. T. , Highlander, S. K. , & Schloss, P. D. (2013). Development of a dual‐index sequencing strategy and curation pipeline for analyzing amplicon sequence data on the miseq illumina sequencing platform. Applied and Environmental Microbiology, 79(17), 5112–5120. 10.1128/AEM.01043-13 23793624 PMC3753973

[ece37172-bib-0027] Lander, T. A. , Harris, S. A. , Cremona, P. J. , & Boshier, D. H. (2019). Impact of habitat loss and fragmentation on reproduction, dispersal and species persistence for an endangered Chilean tree. Conservation Genetics, 20(5), 973–985. 10.1007/s10592-019-01187-z

[ece37172-bib-0028] Letunic, I. , & Bork, P. (2019). Interactive Tree Of Life (iTOL) v4: recent updates and new developments. Nucleic Acids Research, 47(W1), W256–W259. 10.1093/nar/gkz239 30931475 PMC6602468

[ece37172-bib-0029] Li, F. W. , Kuo, L. Y. , Rothfels, C. J. , Ebihara, A. , Chiou, W. L. , Windham, M. D. , & Pryer, K. M. (2011). Rbcl and matk earn two thumbs up as the core DNA barcode for ferns. PLoS One, 6(10), e26597 10.1371/journal.pone.0026597 22028918 PMC3197659

[ece37172-bib-0030] Martínez‐Palacios, A. , Eguiarte, L. E. , & Furnier, G. R. (1999). Genetic diversity of the endangered endemic Agave victoriae‐reginae (Agavaceae) in the Chihuahuan Desert. American Journal of Botany, 86(8), 1093–1098. 10.2307/2656971 10449387

[ece37172-bib-0031] Mendes, R. , Kruijt, M. , De Bruijn, I. , Dekkers, E. , Van Der Voort, M. , Schneider, J. H. M. , Piceno, Y. M. , DeSantis, T. Z. , Andersen, G. L. , Bakker, P. A. H. M. , & Raaijmakers, J. M. (2011). Deciphering the rhizosphere microbiome for disease‐suppressive bacteria. Science, 332(6033), 1097–1100. 10.1126/science.1203980 21551032

[ece37172-bib-0032] Newbold, T. , Hudson, L. N. , Hill, S. L. L. , Contu, S. , Lysenko, I. , Senior, R. A. , Börger, L. , Bennett, D. J. , Choimes, A. , Collen, B. , Day, J. , De Palma, A. , Díaz, S. , Echeverria‐Londoño, S. , Edgar, M. J. , Feldman, A. , Garon, M. , Harrison, M. L. K. , Alhusseini, T. , … Purvis, A. (2015). Global effects of land use on local terrestrial biodiversity. Nature, 520(7545), 45–50. 10.1038/nature14324 25832402

[ece37172-bib-0033] Pardini, R. , Nichols, E. , & Püttker, T. (2018). Biodiversity response to habitat loss and fragmentation In Encyclopedia of the anthropocene. 3, (pp. 229–239). 10.1016/B978-0-12-809665-9.09824-4

[ece37172-bib-0034] Podila, G. K. , Sreedasyam, A. , & Muratet, M. A. (2009). Populus rhizosphere and the ectomycorrhizal interactome. Critical Reviews in Plant Sciences, 28(5), 359–367. 10.1080/07352680903241220

[ece37172-bib-0035] Quast, C. , Pruesse, E. , Yilmaz, P. , Gerken, J. , Schweer, T. , Yarza, P. , Peplies, J. , & Glöckner, F. O. (2013). The SILVA ribosomal RNA gene database project: Improved data processing and web‐based tools. Nucleic Acids Research, 41(D1), D590–D596. 10.1093/nar/gks1219 23193283 PMC3531112

[ece37172-bib-0036] Rodriguez, R. , & Durán, P. (2020). Natural holobiome engineering by using native extreme microbiome to counteract the climate change effects. Frontiers in bioengineering and biotechnology, 8, 568 10.3389/fbioe.2020.00568 32582678 PMC7287022

[ece37172-bib-0037] Roof, J. B. (1971). Shaw's agave is alive and well in San Diego County. Four Seasons, 4(1), 6–8.

[ece37172-bib-0038] Rosenberg, E. , Sharon, G. , Atad, I. , & Zilber‐Rosenberg, I. (2010). The evolution of animals and plants via symbiosis with microorganisms. Environmental Microbiology Reports, 2(4), 500–506. 10.1111/j.1758-2229.2010.00177.x 23766221

[ece37172-bib-0039] Sheng, Y. , & Zhu, L. (2018). Biochar alters microbial community and carbon sequestration potential across different soil pH. Science of the Total Environment, 622–623, 1391–1399. 10.1016/j.scitotenv.2017.11.337 29890604

[ece37172-bib-0040] Sonon, L. S. , Kissel, D. E. , & Vendrell, P. F. (2015). Determining lime requirement using the equilibrium lime buffer capacity.Circular 874, (pp. 1–4). The University of Georgia Cooperative Extension.

[ece37172-bib-0041] Sonon, L. S. , Saha, U. K. , & Kissel, D. E. (2017). Cation exchange capacity and base saturation, Circular 1040, (pp. 1–4). The University of Georgia Cooperative Extension.

[ece37172-bib-0042] Srivastava, D. , Kapoor, R. , Srivastava, S. K. , & Mukerji, K. G. (1996). Vesicular arbuscular mycorrhiza — An overview Concepts in Mycorrhizal Research. Handbook of Vegetation Science. 19/2, (pp. 1–39). 10.1007/978-94-017-1124-1_1

[ece37172-bib-0043] Teng, Y. , Luo, Y. , Sun, M. , Liu, Z. , Li, Z. , & Christie, P. (2010). Effect of bioaugmentation by Paracoccus sp. strain HPD‐2 on the soil microbial community and removal of polycyclic aromatic hydrocarbons from an aged contaminated soil. Bioresource Technology, 101(10), 3437–3443. 10.1016/j.biortech.2009.12.088 20093016

[ece37172-bib-0044] Torrenta, R. , Lacoste, F. , & Villard, M. A. (2018). Loss and fragmentation of mature woodland reduce the habitat niche breadth of forest birds. Landscape Ecology, 33(11), 1865–1879. 10.1007/s10980-018-0718-9

[ece37172-bib-0045] Trivedi, P. , Leach, J. E. , Tringe, S. G. , Sa, T. , & Singh, B. K. (2020). Plant–microbiome interactions: from community assembly to plant health. In Nature reviews microbiology, 18(11), 607–621. 10.1038/s41579-020-0412-1 32788714

[ece37172-bib-0046] Vanderplank, S. E. (2014). A conservation plan for *Agave shawii* subsp. *shawii* (Shaw's Agave, Agavaceae). Rancho Santa Ana Botanic Garden Occasional Publications.

[ece37172-bib-0047] Vanderplank, S. E. , Ezcurra, E. , Delgadillo, J. , Felger, R. , & McDade, L. A. (2014). Conservation challenges in a threatened hotspot: agriculture and plant biodiversity losses in Baja California, Mexico. Biodiversity and Conservation, 23(9), 2173–2182. 10.1007/s10531-014-0711-9

[ece37172-bib-0048] Vanderplank, S. E. , & Lombardo, K. (2015). Shaw's Agave: A short visit. Cactus and Succulent Journal, 87(6), 243–246. 10.2985/015.087.0604

[ece37172-bib-0049] Yilmaz, P. , Parfrey, L. W. , Yarza, P. , Gerken, J. , Pruesse, E. , Quast, C. , Schweer, T. , Peplies, J. , Ludwig, W. , & Glöckner, F. O. (2014). The SILVA and “all‐species Living Tree Project (LTP)” taxonomic frameworks. Nucleic Acids Research, 42(D1), D643–D648. 10.1093/nar/gkt1209 24293649 PMC3965112

[ece37172-bib-0050] Zhalnina, K. , Dias, R. , de Quadros, P. D. , Davis‐Richardson, A. , Camargo, F. A. O. , Clark, I. M. , McGrath, S. P. , Hirsch, P. R. , & Triplett, E. W. (2014). Soil pH determines microbial diversity and composition in the park grass experiment. Microbial Ecology, 69(2), 395–406. 10.1007/s00248-014-0530-2 25395291

