# Supplementary figures and images for: Relative genetic diversity of the rare and endangered Agave shawii ssp. shawii and associated soil microbes within a southern California ecological preserve
Source: Ecol Evol. 2021 Jan 22;11(4):1829–42. doi: 10.1002/ece3.7172 (PMC7882989; doi:10.1002/ece3.7172)

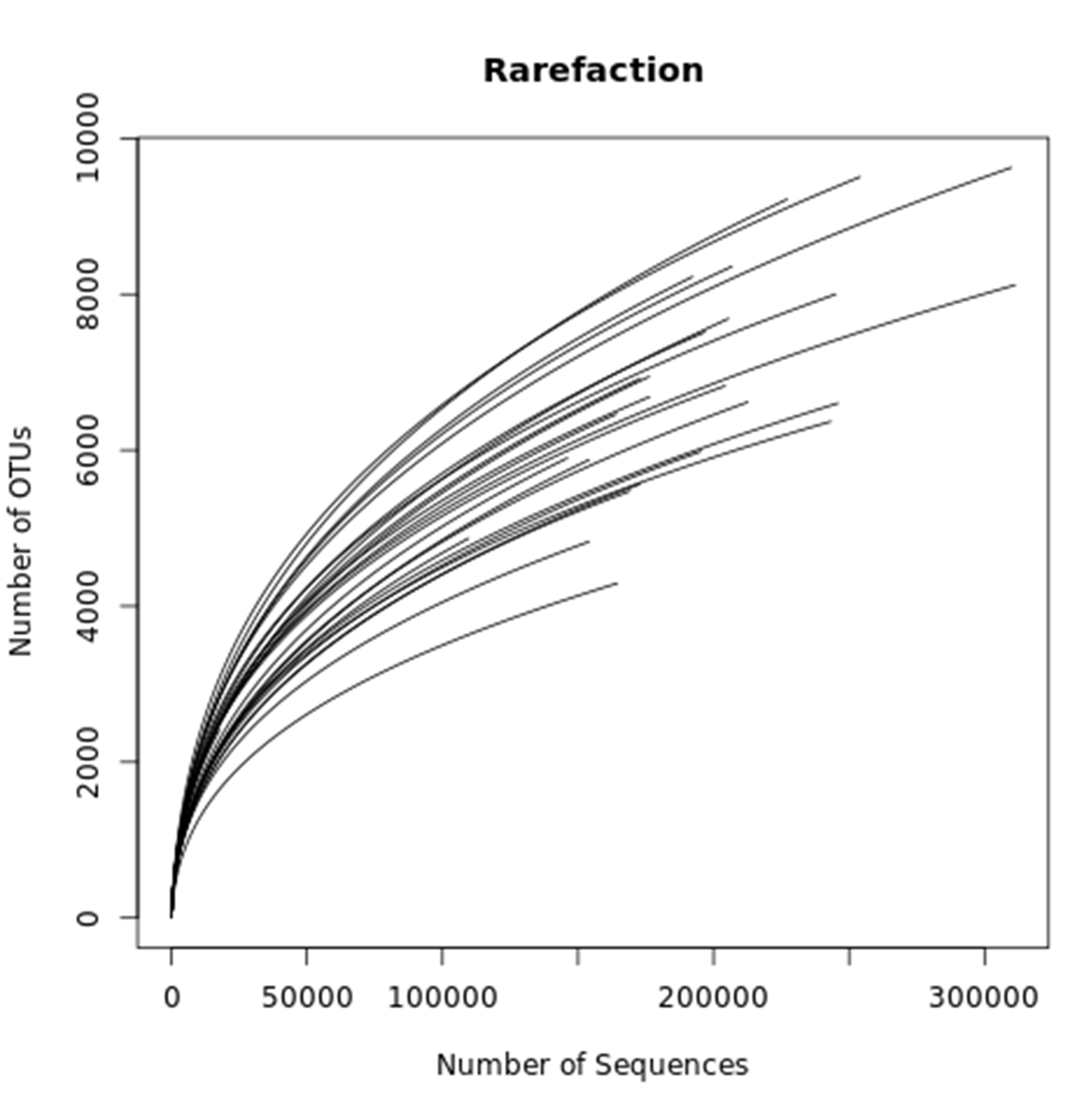

Supplement: Supplementary file 1 — Figure S1 [file ECE3-11-1829-s001.tif]

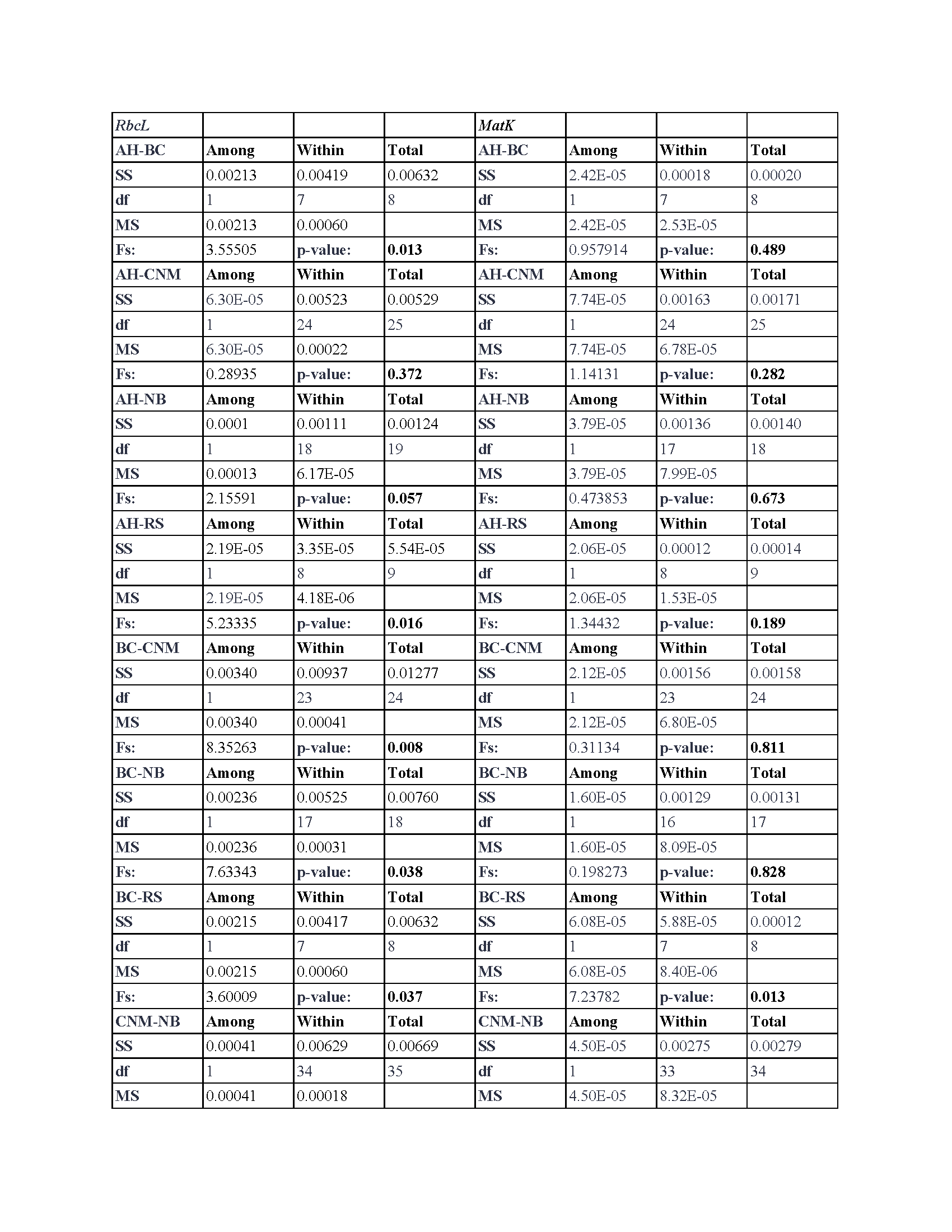

Supplement: Supplementary file 2 — Table S1 [file ECE3-11-1829-s002.tif]

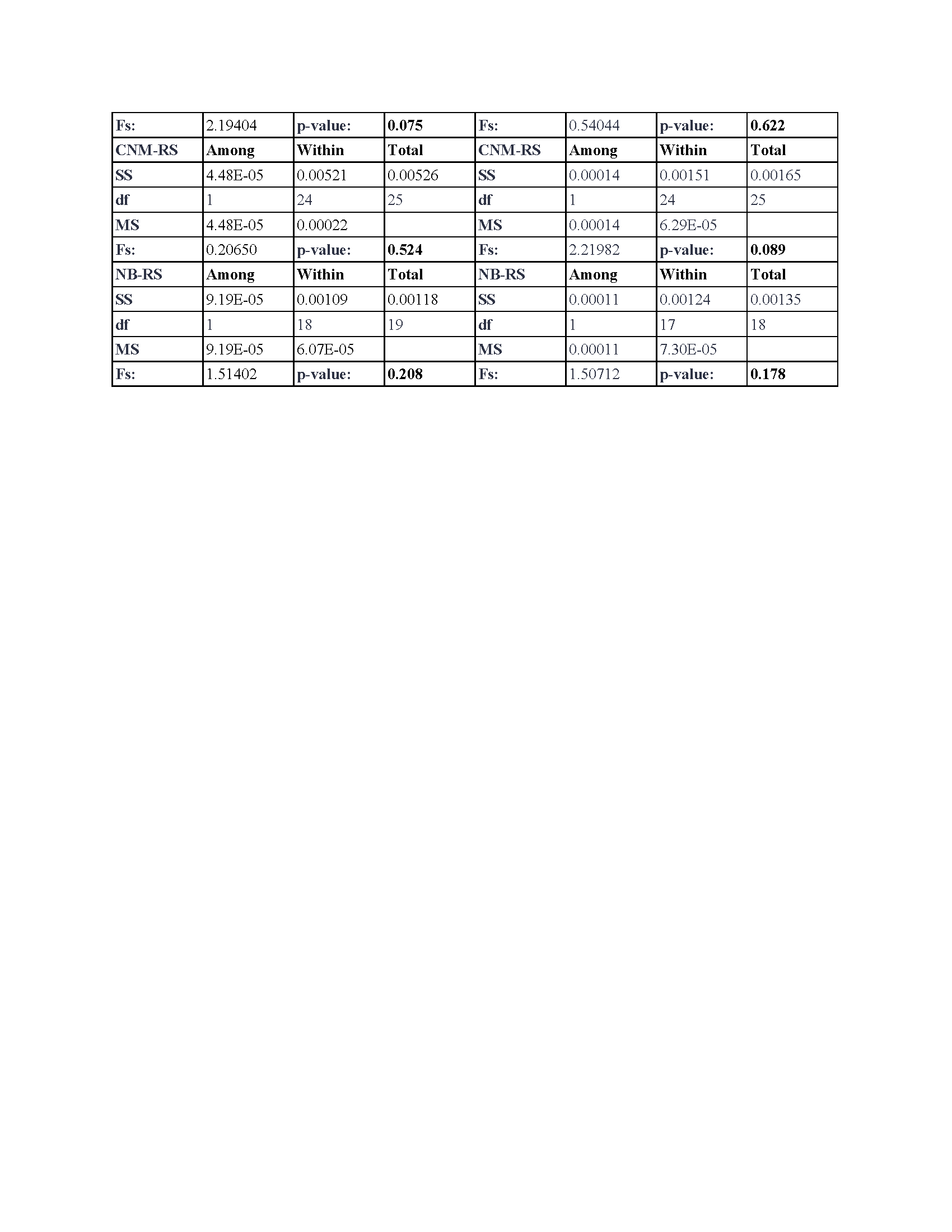

Supplement: Supplementary file 3 — Table S1.1 [file ECE3-11-1829-s003.tif]

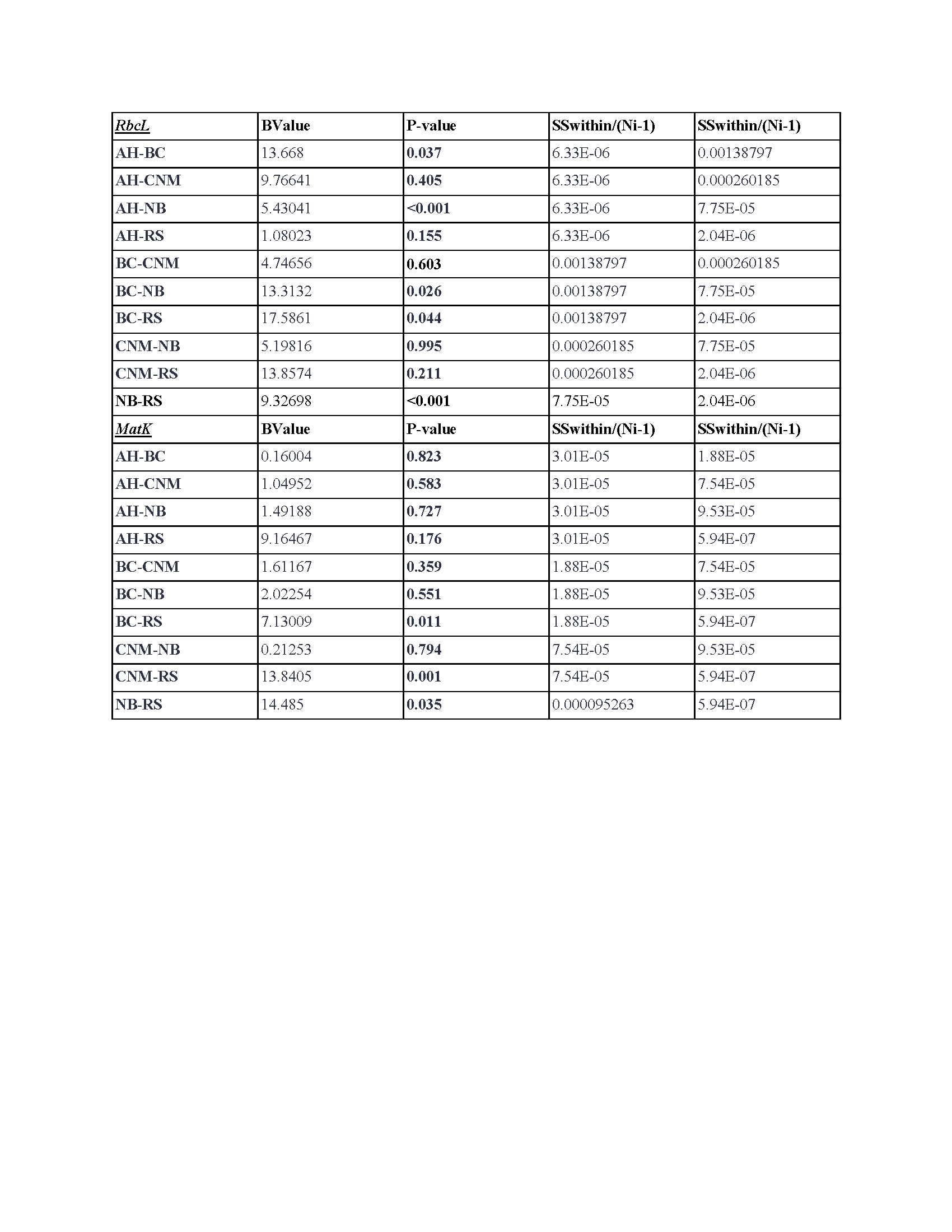

Supplement: Supplementary file 4 — Table S2 [file ECE3-11-1829-s004.tif]

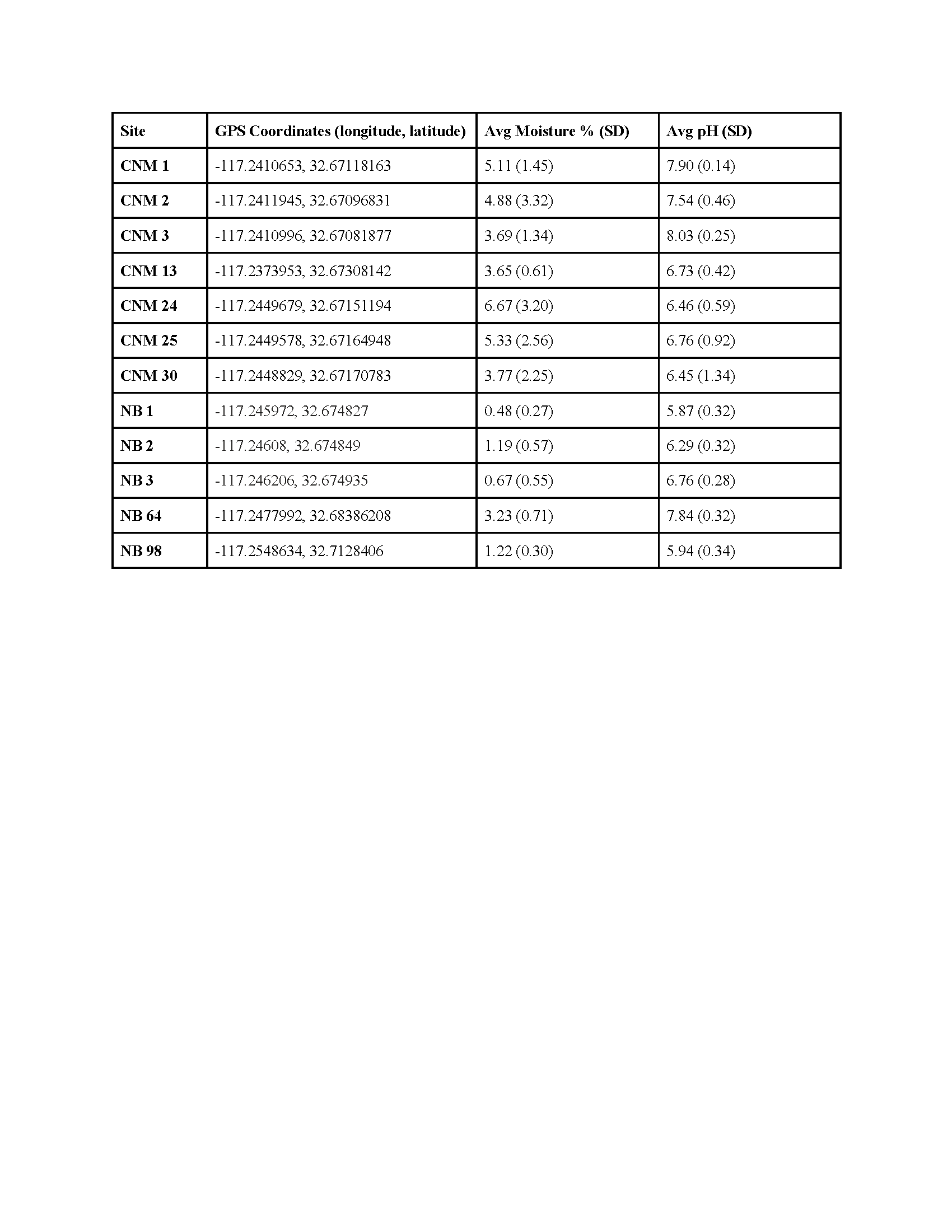

Supplement: Supplementary file 5 — Table S3 [file ECE3-11-1829-s005.tif]
