# Supplementary material for: Relative genetic diversity of the rare and endangered Agave shawii ssp. shawii and associated soil microbes within a southern California ecological preserve
Source: Ecol Evol. 2021 Jan 22;11(4):1829–42. doi: 10.1002/ece3.7172 (PMC7882989; doi:10.1002/ece3.7172)
Supplement: Supplementary file 18 — Supplementary Material [file ECE3-11-1829-s018.docx]

**Supplementary Materials**

| ***RbcL*** |  |  |  | ***MatK*** |  |  |  |
| --- | --- | --- | --- | --- | --- | --- | --- |
| **AH-BC** | **Among** | **Within** | **Total** | **AH-BC** | **Among** | **Within** | **Total** |
| **SS** | 0.00213 | 0.00419 | 0.00632 | **SS** | 2.42E-05 | 0.00018 | 0.00020 |
| **df** | 1 | 7 | 8 | **df** | 1 | 7 | 8 |
| **MS** | 0.00213 | 0.00060 |  | **MS** | 2.42E-05 | 2.53E-05 |  |
| **Fs:** | 3.55505 | **p-value:** | **0.013** | **Fs:** | 0.957914 | **p-value:** | **0.489** |
| **AH-CNM** | **Among** | **Within** | **Total** | **AH-CNM** | **Among** | **Within** | **Total** |
| **SS** | 6.30E-05 | 0.00523 | 0.00529 | **SS** | 7.74E-05 | 0.00163 | 0.00171 |
| **df** | 1 | 24 | 25 | **df** | 1 | 24 | 25 |
| **MS** | 6.30E-05 | 0.00022 |  | **MS** | 7.74E-05 | 6.78E-05 |  |
| **Fs:** | 0.28935 | **p-value:** | **0.372** | **Fs:** | 1.14131 | **p-value:** | **0.282** |
| **AH-NB** | **Among** | **Within** | **Total** | **AH-NB** | **Among** | **Within** | **Total** |
| **SS** | 0.0001 | 0.00111 | 0.00124 | **SS** | 3.79E-05 | 0.00136 | 0.00140 |
| **df** | 1 | 18 | 19 | **df** | 1 | 17 | 18 |
| **MS** | 0.00013 | 6.17E-05 |  | **MS** | 3.79E-05 | 7.99E-05 |  |
| **Fs:** | 2.15591 | **p-value:** | **0.057** | **Fs:** | 0.473853 | **p-value:** | **0.673** |
| **AH-RS** | **Among** | **Within** | **Total** | **AH-RS** | **Among** | **Within** | **Total** |
| **SS** | 2.19E-05 | 3.35E-05 | 5.54E-05 | **SS** | 2.06E-05 | 0.00012 | 0.00014 |
| **df** | 1 | 8 | 9 | **df** | 1 | 8 | 9 |
| **MS** | 2.19E-05 | 4.18E-06 |  | **MS** | 2.06E-05 | 1.53E-05 |  |
| **Fs:** | 5.23335 | **p-value:** | **0.016** | **Fs:** | 1.34432 | **p-value:** | **0.189** |
| **BC-CNM** | **Among** | **Within** | **Total** | **BC-CNM** | **Among** | **Within** | **Total** |
| **SS** | 0.00340 | 0.00937 | 0.01277 | **SS** | 2.12E-05 | 0.00156 | 0.00158 |
| **df** | 1 | 23 | 24 | **df** | 1 | 23 | 24 |
| **MS** | 0.00340 | 0.00041 |  | **MS** | 2.12E-05 | 6.80E-05 |  |
| **Fs:** | 8.35263 | **p-value:** | **0.008** | **Fs:** | 0.31134 | **p-value:** | **0.811** |
| **BC-NB** | **Among** | **Within** | **Total** | **BC-NB** | **Among** | **Within** | **Total** |
| **SS** | 0.00236 | 0.00525 | 0.00760 | **SS** | 1.60E-05 | 0.00129 | 0.00131 |
| **df** | 1 | 17 | 18 | **df** | 1 | 16 | 17 |
| **MS** | 0.00236 | 0.00031 |  | **MS** | 1.60E-05 | 8.09E-05 |  |
| **Fs:** | 7.63343 | **p-value:** | **0.038** | **Fs:** | 0.198273 | **p-value:** | **0.828** |
| **BC-RS** | **Among** | **Within** | **Total** | **BC-RS** | **Among** | **Within** | **Total** |
| **SS** | 0.00215 | 0.00417 | 0.00632 | **SS** | 6.08E-05 | 5.88E-05 | 0.00012 |
| **df** | 1 | 7 | 8 | **df** | 1 | 7 | 8 |
| **MS** | 0.00215 | 0.00060 |  | **MS** | 6.08E-05 | 8.40E-06 |  |
| **Fs:** | 3.60009 | **p-value:** | **0.037** | **Fs:** | 7.23782 | **p-value:** | **0.013** |
| **CNM-NB** | **Among** | **Within** | **Total** | **CNM-NB** | **Among** | **Within** | **Total** |
| **SS** | 0.00041 | 0.00629 | 0.00669 | **SS** | 4.50E-05 | 0.00275 | 0.00279 |
| **df** | 1 | 34 | 35 | **df** | 1 | 33 | 34 |
| **MS** | 0.00041 | 0.00018 |  | **MS** | 4.50E-05 | 8.32E-05 |  |
| **Fs:** | 2.19404 | **p-value:** | **0.075** | **Fs:** | 0.54044 | **p-value:** | **0.622** |
| **CNM-RS** | **Among** | **Within** | **Total** | **CNM-RS** | **Among** | **Within** | **Total** |
| **SS** | 4.48E-05 | 0.00521 | 0.00526 | **SS** | 0.00014 | 0.00151 | 0.00165 |
| **df** | 1 | 24 | 25 | **df** | 1 | 24 | 25 |
| **MS** | 4.48E-05 | 0.00022 |  | **MS** | 0.00014 | 6.29E-05 |  |
| **Fs:** | 0.20650 | **p-value:** | **0.524** | **Fs:** | 2.21982 | **p-value:** | **0.089** |
| **NB-RS** | **Among** | **Within** | **Total** | **NB-RS** | **Among** | **Within** | **Total** |
| **SS** | 9.19E-05 | 0.00109 | 0.00118 | **SS** | 0.00011 | 0.00124 | 0.00135 |
| **df** | 1 | 18 | 19 | **df** | 1 | 17 | 18 |
| **MS** | 9.19E-05 | 6.07E-05 |  | **MS** | 0.00011 | 7.30E-05 |  |
| **Fs:** | 1.51402 | **p-value:** | **0.208** | **Fs:** | 1.50712 | **p-value:** | **0.178** |

**Table S1. AMOVA analysis for both pooled Shaw’s Agave *rbcL* and *matK* barcode sequence samples between coastal agave sites.** Significant site differences suggest distinct genetic diversity between the two sites. CNM = Cabrillo National Monument, NB = Point Loma Naval Base, BC = Border Crossing, RS = Rosarito, MX, AH = Arroyo Hondo, MX.

| ***RbcL*** | **BValue** | **P-value** | **SSwithin/(Ni-1)** | **SSwithin/(Ni-1)** |
| --- | --- | --- | --- | --- |
| **AH-BC** | 13.668 | **0.037** | 6.33E-06 | 0.00138797 |
| **AH-CNM** | 9.76641 | **0.405** | 6.33E-06 | 0.000260185 |
| **AH-NB** | 5.43041 | **<0.001** | 6.33E-06 | 7.75E-05 |
| **AH-RS** | 1.08023 | **0.155** | 6.33E-06 | 2.04E-06 |
| **BC-CNM** | 4.74656 | **0.603** | 0.00138797 | 0.000260185 |
| **BC-NB** | 13.3132 | **0.026** | 0.00138797 | 7.75E-05 |
| **BC-RS** | 17.5861 | **0.044** | 0.00138797 | 2.04E-06 |
| **CNM-NB** | 5.19816 | **0.995** | 0.000260185 | 7.75E-05 |
| **CNM-RS** | 13.8574 | **0.211** | 0.000260185 | 2.04E-06 |
| **NB-RS** | 9.32698 | **<0.001** | 7.75E-05 | 2.04E-06 |
| ***MatK*** | **BValue** | **P-value** | **SSwithin/(Ni-1)** | **SSwithin/(Ni-1)** |
| **AH-BC** | 0.16004 | **0.823** | 3.01E-05 | 1.88E-05 |
| **AH-CNM** | 1.04952 | **0.583** | 3.01E-05 | 7.54E-05 |
| **AH-NB** | 1.49188 | **0.727** | 3.01E-05 | 9.53E-05 |
| **AH-RS** | 9.16467 | **0.176** | 3.01E-05 | 5.94E-07 |
| **BC-CNM** | 1.61167 | **0.359** | 1.88E-05 | 7.54E-05 |
| **BC-NB** | 2.02254 | **0.551** | 1.88E-05 | 9.53E-05 |
| **BC-RS** | 7.13009 | **0.011** | 1.88E-05 | 5.94E-07 |
| **CNM-NB** | 0.21253 | **0.794** | 7.54E-05 | 9.53E-05 |
| **CNM-RS** | 13.8405 | **0.001** | 7.54E-05 | 5.94E-07 |
| **NB-RS** | 14.485 | **0.035** | 0.000095263 | 5.94E-07 |

**Table S2. HOMOVA analysis for both pooled Shaw’s Agave r*bcL* and *matK* barcode sequence samples between coastal agave sites.** Significant site differences suggest the genetic diversity between the sites are non-homogenous. CNM = Cabrillo National Monument, NB = Point Loma Naval Base, BC = Border Crossing, RS = Rosarito, MX, AH = Arroyo Hondo, MX.

| **Site** | **GPS Coordinates (longitude, latitude)** | **Avg Moisture % (SD)** | **Avg pH (SD)** |
| --- | --- | --- | --- |
| **CNM 1** | -117.2410653, 32.67118163 | 5.11 (1.45) | 7.90 (0.14) |
| **CNM 2** | -117.2411945, 32.67096831 | 4.88 (3.32) | 7.54 (0.46) |
| **CNM 3** | -117.2410996, 32.67081877 | 3.69 (1.34) | 8.03 (0.25) |
| **CNM 13** | -117.2373953, 32.67308142 | 3.65 (0.61) | 6.73 (0.42) |
| **CNM 24** | -117.2449679, 32.67151194 | 6.67 (3.20) | 6.46 (0.59) |
| **CNM 25** | -117.2449578, 32.67164948 | 5.33 (2.56) | 6.76 (0.92) |
| **CNM 30** | -117.2448829, 32.67170783 | 3.77 (2.25) | 6.45 (1.34) |
| **NB 1** | -117.245972, 32.674827 | 0.48 (0.27) | 5.87 (0.32) |
| **NB 2** | -117.24608, 32.674849 | 1.19 (0.57) | 6.29 (0.32) |
| **NB 3** | -117.246206, 32.674935 | 0.67 (0.55) | 6.76 (0.28) |
| **NB 64** | -117.2477992, 32.68386208 | 3.23 (0.71) | 7.84 (0.32) |
| **NB 98** | -117.2548634, 32.7128406 | 1.22 (0.30) | 5.94 (0.34) |

**Table S3. Cabrillo National Monument (CNM) samples have significantly higher moisture (p < 0.0001) and more basic pH than Point Loma Naval Base (NB) samples (p < 0.05).** Physical properties of Shaw’s Agave soil. Samples were collected from sites surrounding 12 different Shaw’s Agave individuals within Point Loma (7 from Cabrillo National Monument, 5 from the Point Loma Naval Base). Three sample replicates from each site (n = 3) were pooled to calculate moisture content and pH.

|  | CNM 1 | CNM 2 | CNM 3 | CNM 13 | CNM 24 | CNM 25 | CNM 30 | NB 1 | NB 2 | NB 3 | NB 64 | NB 98 |
| --- | --- | --- | --- | --- | --- | --- | --- | --- | --- | --- | --- | --- |
| LBC (ppm CaCO_3_/pH) | 300 | 392 | 303 | 224 | 340 | 402 | 400 | 256 | 224 | 292 | 334 | 244 |
| LBCeq (ppm CaCO_3_/pH) | 870 | 1137 | 879 | 634 | 986 | 1166 | 1160 | 742 | 634 | 847 | 969 | 707 |
| pH | 8.09 | 7.97 | 7.98 | 6.55 | 6.65 | 6.67 | 5.96 | 6.16 | 6.05 | 7.09 | 7.87 | 5.91 |
| Base saturation (%) | 100.0 | 100.0 | 100.0 | 94.9 | 94.5 | 95.3 | 83.8 | 85.4 | 83.6 | 100.0 | 100.0 | 79.9 |
| CEC (meq/100g) | 22.88 | 31.86 | 26.67 | 11.23 | 12.46 | 16.30 | 14.91 | 8.53 | 7.37 | 13.20 | 19.57 | 7.69 |
| Ca (mg/kg) | 3498 | 4683 | 3795 | 537 | 795 | 716 | 1063 | 672 | 583 | 1050 | 2025 | 692 |
| Cd (mg/kg) | 0.05 | 0.07 | 0.06 | <0.04 | 0.11 | 0.13 | 0.14 | 0.05 | 0.06 | 0.06 | 0.06 | 0.08 |
| Cr (mg/kg) | 0.05 | 0.08 | 0.08 | 0.04 | <0.05 | <0.05 | <0.05 | <0.04 | <0.04 | <0.04 | <0.04 | <0.04 |
| Cu (mg/kg) | 0.67 | 0.52 | 0.76 | 0.35 | 0.25 | 0.42 | <0.24 | <0.18 | <0.20 | <0.19 | 0.62 | <0.19 |
| Fe (mg/kg) | 42.63 | 30.38 | 43.39 | 61.81 | 23.42 | 30.87 | 27.89 | 26.16 | 20.65 | 19.08 | 38.23 | 21.85 |
| K (mg/kg) | 258.8 | 652.2 | 496.5 | 118.8 | 204.6 | 280.8 | 186.7 | 170.5 | 106.9 | 331.8 | 244.9 | 207.0 |
| Mg (mg/kg) | 531.7 | 687.7 | 705.4 | 785.1 | 724.1 | 950.2 | 621.9 | 280.1 | 261.7 | 724.8 | 893.6 | 162.2 |
| Mn (mg/kg) | 52.42 | 67.92 | 65.41 | 12.88 | 46.89 | 47.38 | 65.24 | 41.26 | 42.83 | 35.46 | 31.89 | 29.64 |
| Mo (mg/kg) | <0.05 | <0.05 | <0.04 | <0.04 | <0.05 | <0.05 | <0.05 | <0.04 | <0.04 | <0.04 | <0.04 | <0.04 |
| Na (mg/kg) | 67.8 | 239.1 | 124.7 | 258.3 | 284.7 | 761.4 | 349.2 | 264.6 | 182.8 | 244.6 | 314.4 | 184.7 |
| Ni (mg/kg) | 0.32 | 0.40 | 0.39 | 0.27 | 0.49 | 0.50 | 0.37 | 0.38 | 0.33 | 0.33 | 0.65 | 0.25 |
| P (mg/kg) | 158.6 | 193.6 | 203.6 | 11.4 | 40.2 | 50.8 | 40.1 | 31.2 | 21.6 | 71.7 | 48.0 | 43.7 |
| Pb (mg/kg) | 0.11 | 0.14 | 0.16 | 0.03 | 0.53 | 0.44 | 0.64 | 0.13 | 0.14 | 0.22 | 0.08 | 0.28 |
| Zn (mg/kg) | 4.72 | 6.23 | 6.08 | 1.99 | 6.68 | 9.88 | 8.77 | 4.13 | 3.92 | 4.93 | 6.72 | 4.80 |
| NO_3_-N (mg/kg) | 4.26 | 8.93 | 5.88 | 3.65 | 5.90 | 7.10 | 6.70 | 7.22 | 4.68 | 16.05 | 7.35 | 12.51 |
| OM (%) | 2.55 | 4.46 | 2.43 | 1.47 | 3.06 | 3.46 | 3.74 | 1.95 | 2.42 | 2.86 | 2.92 | 1.68 |

**Table S4. Chemical analysis of soil samples from Cabrillo National Monument (CNM) and Point Loma Naval Base (NB).** LBC = lime buffer capacity, LBCeq = equilibrium lime buffer capacity, CEC = cation exchange capacity, and OM = organic matters. A value with a less-than sign indicates relatively low amount that falls below the detection limit.

| **Sample** | **Shannon’s Evenness (SD)** | **Shannon’s Richness (SD)** | **Shannon’s Index (SD)** | **Simpson’s Evenness (SD)** | **Simpson’s Index (SD)** |
| --- | --- | --- | --- | --- | --- |
| **CNM 1** | 0.7319 (0.0392) | 6414.000 (66.468) | 6.4158 (0.3515) | 0.0260 (0.0159) | 0.0074 (0.0046) |
| **CNM 3** | 0.7513 | 6916.0000 | 6.6425 | 0.0316 | 0.0046 |
| **CNM 13** | 0.7321 (0.0215) | 6150.3333 (1426.3395) | 6.3720 (0.1367) | 0.0287 (0.0105) | 0.0062(0.0016) |
| **CNM 25** | 0.7477 (0.0098) | 8580.0000 (906.0535) | 6.7691 (0.1359) | 0.0293 (0.0017) | 0.0040 (0.0006) |
| **CNM 30** | 0.7478 (0.0259) | 7648.3333 (1610.4485) | 6.6784 (0.3370) | 0.0310 (0.0086) | 0.0045 (0.0015) |
| **NB 1** | 0.7334 (0.0204) | 6421.6667 (382.9104) | 6.4299 (0.2119) | 0.0270 (0.0026) | 0.0058 (0.0008) |
| **NB 2** | 0.7173 (0.0382) | 5600.0000 (1325.8081) | 6.1832 (0.4972) | 0.0284 (0.0106) | 0.0079 (0.0055) |
| **NB 3** | 0.75963(0.0461) | 5198.6667 (2987.0806) | 6.3893 (0.1924) | 0.0390 (0.0240) | 0.0064 (0.0006) |
| **NB 64** | 0.7358 | 8225.0000 | 6.6329 | 0.0185 | 0.0066 |
| **NB 98** | 0.7327 (0.0043) | 7400.6667 (1874.4814) | 6.5120 (0.2307) | 0.0252 (0.0049) | 0.0056 (0.0005) |
| **CNM (all sites)** | 0.7421 (0.0094) | 7141.7333 (985.9868) | 6.5756 (0.1728) | 0.0293 (0.0022) | 0.0054 (0.0014) |
| **NB (all sites)** | 0.7358 (0.0187) | 6569.2000 (1087.5925) | 6.4295 (0.1436) | 0.0276 (0.0096) | 0.0065 (0.0024) |

**Table S5. Simpson’s and Shannon’s diversity calculators across all sampled Shaw’s Agave soil samples for microbial diversity by 16S rRNA analysis.** Bacterial diversity index values calculated by Simpson’s and Shannon’s diversity index, evenness, and richness across all Cabrillo National Monument (CNM) and Naval Base (NB) soil samples. CNM 3 and NB 64 did not have replicates in the 16S rRNA analysis. There is no statistically significant difference between the two sites.

**
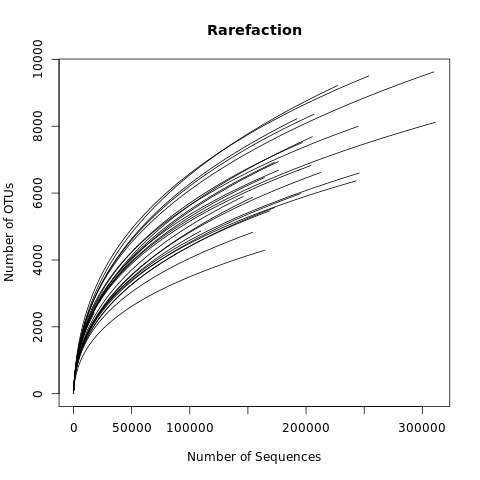
**

**Figure S1. Rarefaction curves across all sample sites calculated by sob (observed species richness) demonstrate adequate sampling.** Microbial sequences across all CNM and NB soil samples have a representative survey of species as operational taxonomic unit (OTU) discovery flattens as the number of sequences sampled increases.

| Sample ID | | Number of species | | | Shared species | Shared species | Shared species | Shared species |
| --- | --- | --- | --- | --- | --- | --- | --- | --- |
| Site | Cluster | A | B | C | A + B | A + C | B + C | A + B + C |
| CNM | 13 | 9872 | 12020 | 15744 | 3907 | 3875 | 4329 | 2836 |
| CNM | 25 | 15200 | 14855 | 18380 | 3895 | 5965 | 4027 | 2832 |
| CNM | 30 | 12737 | 13032 | 17457 | 3347 | 4611 | 3927 | 2408 |
| NB | 1 | 12454 | 13150 | 12211 | 4431 | 3695 | 4737 | 3025 |
| NB | 2 | 14143 | 11618 | 8598 | 4609 | 3074 | 3384 | 2683 |
| NB | 3 | 15724 | 5128 | 9593 | 2185 | 3039 | 1916 | 1498 |
| NB | 98 | 14232 | 17632 | 10549 | 5879 | 4226 | 4365 | 3337 |

**Table S6. Shared number of soil microbial species within Point Loma Shaw’s Agave sample sites computed via abundance-based coverage estimator (ACE) of species richness.** Species richness between CNM (Cabrillo National Monument) and NB (Point Loma Naval Base) compared across sample sites (number 1-98) and compared within three biological replicates (A/B/C). Computed number of shared species rounded to the nearest integer value.

| **CNM 13ABC-CNM 1AC** | **Among** | **Within** | **Total** |
| --- | --- | --- | --- |
| **SS** | 0.396185 | 0.411592 | 0.807777 |
| **df** | 1 | 3 | 4 |
| **MS** | 0.396185 | 0.137197 |  |
| **Fs:** | 2.8877 | **p-value:** | **0.101** |
| **CNM 13ABC-CNM 25ABC** | **Among** | **Within** | **Total** |
| **SS** | 0.35437 | 0.656838 | 1.01121 |
| **df** | 1 | 4 | 5 |
| **MS** | 0.35437 | 0.16421 |  |
| **Fs:** | 2.15804 | **p-value:** | **0.087** |
| **CNM 13ABC-CNM 30ABC** | **Among** | **Within** | **Total** |
| **SS** | 0.346563 | 0.701814 | 1.04838 |
| **df** | 1 | 4 | 5 |
| **MS** | 0.346563 | 0.175454 |  |
| **Fs:** | 1.97524 | **p-value:** | **0.108** |
| **CNM 13ABC-CNM 3B** | **Among** | **Within** | **Total** |
| **SS** | 0.324073 | 0.253881 | 0.577954 |
| **df** | 1 | 2 | 3 |
| **MS** | 0.324073 | 0.126941 |  |
| **Fs:** | 2.55295 | **p-value:** | **0.242** |
| **CNM 13ABC-NB 1ABC** | **Among** | **Within** | **Total** |
| **SS** | 0.30733 | 0.481135 | 0.788465 |
| **df** | 1 | 4 | 5 |
| **MS** | 0.30733 | 0.120284 |  |
| **Fs:** | 2.55504 | **p-value:** | **0.086** |
| **CNM 13ABC-NB 2ABC** | **Among** | **Within** | **Total** |
| **SS** | 0.301648 | 0.53188 | 0.833528 |
| **df** | 1 | 4 | 5 |
| **MS** | 0.301648 | 0.13297 |  |
| **Fs:** | 2.26854 | **p-value:** | **0.096** |
| **CNM 13ABC-NB 3ABC** | **Among** | **Within** | **Total** |
| **SS** | 0.388601 | 0.729564 | 1.11817 |
| **df** | 1 | 4 | 5 |
| **MS** | 0.388601 | 0.182391 |  |
| **Fs:** | 2.13059 | **p-value:** | **0.091** |
| **CNM 13ABC-NB 64C** | **Among** | **Within** | **Total** |
| **SS** | 0.260758 | 0.253881 | 0.514639 |
| **df** | 1 | 2 | 3 |
| **MS** | 0.260758 | 0.126941 |  |
| **Fs:** | 2.05417 | **p-value:** | **0.279** |
| **CNM 13ABC-NB 98ABC** | **Among** | **Within** | **Total** |
| **SS** | 0.272196 | 0.512906 | 0.785101 |
| **df** | 1 | 4 | 5 |
| **MS** | 0.272196 | 0.128226 |  |
| **Fs:** | 2.12277 | **p-value:** | **0.105** |
| **CNM 1AC-CNM 25ABC** | **Among** | **Within** | **Total** |
| **SS** | 0.321051 | 0.560668 | 0.881719 |
| **df** | 1 | 3 | 4 |
| **MS** | 0.321051 | 0.186889 |  |
| **Fs:** | 1.71787 | **p-value:** | **0.202** |
| **CNM 1AC-CNM 30ABC** | **Among** | **Within** | **Total** |
| **SS** | 0.306346 | 0.605644 | 0.91199 |
| **df** | 1 | 3 | 4 |
| **MS** | 0.306346 | 0.201881 |  |
| **Fs:** | 1.51745 | **p-value:** | **0.206** |
| **CNM 1AC-CNM 3B** | **Among** | **Within** | **Total** |
| **SS** | 0.110794 | 0.157711 | 0.268505 |
| **df** | 1 | 1 | 2 |
| **MS** | 0.110794 | 0.157711 |  |
| **Fs:** | 0.702517 | **p-value:** | **1** |
| **CNM 1AC-NB 1ABC** | **Among** | **Within** | **Total** |
| **SS** | 0.421105 | 0.384965 | 0.80607 |
| **df** | 1 | 3 | 4 |
| **MS** | 0.421105 | 0.128322 |  |
| **Fs:** | 3.28164 | **p-value:** | **0.112** |
| **CNM 1AC-NB 2ABC** | **Among** | **Within** | **Total** |
| **SS** | 0.49941 | 0.43571 | 0.935119 |
| **df** | 1 | 3 | 4 |
| **MS** | 0.49941 | 0.145237 |  |
| **Fs:** | 3.43859 | **p-value:** | **0.103** |
| **CNM 1AC-NB 3ABC** | **Among** | **Within** | **Total** |
| **SS** | 0.334184 | 0.633394 | 0.967578 |
| **df** | 1 | 3 | 4 |
| **MS** | 0.334184 | 0.211131 |  |
| **Fs:** | 1.58283 | **p-value:** | **0.209** |
| **CNM 1AC-NB 64C** | **Among** | **Within** | **Total** |
| **SS** | 0.175163 | 0.157711 | 0.332873 |
| **df** | 1 | 1 | 2 |
| **MS** | 0.175163 | 0.157711 |  |
| **Fs:** | 1.11066 | **p-value:** | **0.657** |
| **CNM 1AC-NB 98ABC** | **Among** | **Within** | **Total** |
| **SS** | 0.336532 | 0.416735 | 0.753267 |
| **df** | 1 | 3 | 4 |
| **MS** | 0.336532 | 0.138912 |  |
| **Fs:** | 2.42263 | **p-value:** | **0.089** |
| **CNM 25ABC-CNM 30ABC** | **Among** | **Within** | **Total** |
| **SS** | 0.127891 | 0.85089 | 0.978781 |
| **df** | 1 | 4 | 5 |
| **MS** | 0.127891 | 0.212723 |  |
| **Fs:** | 0.60121 | **p-value:** | **1** |
| **CNM 25ABC-CNM 3B** | **Among** | **Within** | **Total** |
| **SS** | 0.240493 | 0.402957 | 0.64345 |
| **df** | 1 | 2 | 3 |
| **MS** | 0.240493 | 0.201479 |  |
| **Fs:** | 1.19364 | **p-value:** | **0.468** |
| **CNM 25ABC-NB 1ABC** | **Among** | **Within** | **Total** |
| **SS** | 0.217885 | 0.630211 | 0.848097 |
| **df** | 1 | 4 | 5 |
| **MS** | 0.217885 | 0.157553 |  |
| **Fs:** | 1.38294 | **p-value:** | **0.116** |
| **CNM 25ABC-NB 2ABC** | **Among** | **Within** | **Total** |
| **SS** | 0.308831 | 0.680956 | 0.989787 |
| **df** | 1 | 4 | 5 |
| **MS** | 0.308831 | 0.170239 |  |
| **Fs:** | 1.8141 | **p-value:** | **0.096** |
| **CNM 25ABC-NB 3ABC** | **Among** | **Within** | **Total** |
| **SS** | 0.187149 | 0.87864 | 1.06579 |
| **df** | 1 | 4 | 5 |
| **MS** | 0.187149 | 0.21966 |  |
| **Fs:** | 0.851995 | **p-value:** | **0.713** |
| **CNM 25ABC-NB 64C** | **Among** | **Within** | **Total** |
| **SS** | 0.27779 | 0.402957 | 0.680747 |
| **df** | 1 | 2 | 3 |
| **MS** | 0.27779 | 0.201479 |  |
| **Fs:** | 1.37876 | **p-value:** | **0.233** |
| **CNM 25ABC-NB 98ABC** | **Among** | **Within** | **Total** |
| **SS** | 0.23322 | 0.661982 | 0.895202 |
| **df** | 1 | 4 | 5 |
| **MS** | 0.23322 | 0.165495 |  |
| **Fs:** | 1.40922 | **p-value:** | **0.187** |
| **CNM 30ABC-CNM 3B** | **Among** | **Within** | **Total** |
| **SS** | 0.235931 | 0.447933 | 0.683864 |
| **df** | 1 | 2 | 3 |
| **MS** | 0.235931 | 0.223967 |  |
| **Fs:** | 1.05342 | **p-value:** | **0.504** |
| **CNM 30ABC-NB 1ABC** | **Among** | **Within** | **Total** |
| **SS** | 0.214807 | 0.675187 | 0.889995 |
| **df** | 1 | 4 | 5 |
| **MS** | 0.214807 | 0.168797 |  |
| **Fs:** | 1.27258 | **p-value:** | **0.21** |
| **CNM 30ABC-NB 2ABC** | **Among** | **Within** | **Total** |
| **SS** | 0.296054 | 0.725932 | 1.02199 |
| **df** | 1 | 4 | 5 |
| **MS** | 0.296054 | 0.181483 |  |
| **Fs:** | 1.6313 | **p-value:** | **0.194** |
| **CNM 30ABC-NB 3ABC** | **Among** | **Within** | **Total** |
| **SS** | 0.198929 | 0.923616 | 1.12255 |
| **df** | 1 | 4 | 5 |
| **MS** | 0.198929 | 0.230904 |  |
| **Fs:** | 0.861522 | **p-value:** | **0.79** |
| **CNM 30ABC-NB 64C** | **Among** | **Within** | **Total** |
| **SS** | 0.265455 | 0.447933 | 0.713388 |
| **df** | 1 | 2 | 3 |
| **MS** | 0.265455 | 0.223967 |  |
| **Fs:** | 1.18524 | **p-value:** | **0.51** |
| **CNM 30ABC-NB 98ABC** | **Among** | **Within** | **Total** |
| **SS** | 0.211115 | 0.706958 | 0.918072 |
| **df** | 1 | 4 | 5 |
| **MS** | 0.211115 | 0.176739 |  |
| **Fs:** | 1.1945 | **p-value:** | **0.32** |
| **CNM 3B-NB 1ABC** | **Among** | **Within** | **Total** |
| **SS** | 0.324141 | 0.227254 | 0.551395 |
| **df** | 1 | 2 | 3 |
| **MS** | 0.324141 | 0.113627 |  |
| **Fs:** | 2.85267 | **p-value:** | **0.274** |
| **CNM 3B-NB 2ABC** | **Among** | **Within** | **Total** |
| **SS** | 0.386577 | 0.277999 | 0.664576 |
| **df** | 1 | 2 | 3 |
| **MS** | 0.386577 | 0.139 |  |
| **Fs:** | 2.78114 | **p-value:** | **0.237** |
| **CNM 3B-NB 3ABC** | **Among** | **Within** | **Total** |
| **SS** | 0.246607 | 0.475683 | 0.72229 |
| **df** | 1 | 2 | 3 |
| **MS** | 0.246607 | 0.237842 |  |
| **Fs:** | 1.03685 | **p-value:** | **0.512** |
| **CNM 3B-NB 98ABC** | **Among** | **Within** | **Total** |
| **SS** | 0.256114 | 0.259025 | 0.515138 |
| **df** | 1 | 2 | 3 |
| **MS** | 0.256114 | 0.129512 |  |
| **Fs:** | 1.97752 | **p-value:** | **0.278** |
| **NB 1ABC-NB 2ABC** | **Among** | **Within** | **Total** |
| **SS** | 0.191157 | 0.505253 | 0.69641 |
| **df** | 1 | 4 | 5 |
| **MS** | 0.191157 | 0.126313 |  |
| **Fs:** | 1.51335 | **p-value:** | **0.113** |
| **NB 1ABC-NB 3ABC** | **Among** | **Within** | **Total** |
| **SS** | 0.203905 | 0.702937 | 0.906842 |
| **df** | 1 | 4 | 5 |
| **MS** | 0.203905 | 0.175734 |  |
| **Fs:** | 1.1603 | **p-value:** | **0.323** |
| **NB 1ABC-NB 64C** | **Among** | **Within** | **Total** |
| **SS** | 0.349526 | 0.227254 | 0.57678 |
| **df** | 1 | 2 | 3 |
| **MS** | 0.349526 | 0.113627 |  |
| **Fs:** | 3.07608 | **p-value:** | **0.261** |
| **NB 1ABC-NB 98ABC** | **Among** | **Within** | **Total** |
| **SS** | 0.233655 | 0.486279 | 0.719934 |
| **df** | 1 | 4 | 5 |
| **MS** | 0.233655 | 0.12157 |  |
| **Fs:** | 1.92199 | **p-value:** | **0.102** |
| **NB 2ABC-NB 3ABC** | **Among** | **Within** | **Total** |
| **SS** | 0.333191 | 0.753682 | 1.08687 |
| **df** | 1 | 4 | 5 |
| **MS** | 0.333191 | 0.188421 |  |
| **Fs:** | 1.76834 | **p-value:** | **0.119** |
| **NB 2ABC-NB 64C** | **Among** | **Within** | **Total** |
| **SS** | 0.406023 | 0.277999 | 0.684022 |
| **df** | 1 | 2 | 3 |
| **MS** | 0.406023 | 0.139 |  |
| **Fs:** | 2.92104 | **p-value:** | **0.257** |
| **NB 2ABC-NB 98ABC** | **Among** | **Within** | **Total** |
| **SS** | 0.309391 | 0.537024 | 0.846414 |
| **df** | 1 | 4 | 5 |
| **MS** | 0.309391 | 0.134256 |  |
| **Fs:** | 2.30448 | **p-value:** | **0.105** |
| **NB 3ABC-NB 64C** | **Among** | **Within** | **Total** |
| **SS** | 0.314216 | 0.475683 | 0.789899 |
| **df** | 1 | 2 | 3 |
| **MS** | 0.314216 | 0.237842 |  |
| **Fs:** | 1.32111 | **p-value:** | **0.255** |
| **NB 3ABC-NB 98ABC** | **Among** | **Within** | **Total** |
| **SS** | 0.254989 | 0.734708 | 0.989697 |
| **df** | 1 | 4 | 5 |
| **MS** | 0.254989 | 0.183677 |  |
| **Fs:** | 1.38825 | **p-value:** | **0.183** |
| **NB 64C-NB 98ABC** | **Among** | **Within** | **Total** |
| **SS** | 0.271601 | 0.259025 | 0.530625 |
| **df** | 1 | 2 | 3 |
| **MS** | 0.271601 | 0.129512 |  |
| **Fs:** | 2.0971 | **p-value:** | **0.257** |

**Table S7. PERMANOVA calculation for soil microbial structural diversity across all agave samples sites.** Replicate agave 16S rRNA sample data were grouped together by sample site. All calculations are based on 16S microbial operational taxonomic unit (OTU) clustering. SS = sum of squares, df = degrees of freedom, MS = mean sum of squares, and Fs = F-statistic. There is no significant difference for any pairwise comparison. Comparison between CNM 3 and NB 64 are excluded as they do not have replicates.
